# Diagnostic imaging of adnexal lesions in pregnancy: systematic review

**DOI:** 10.1002/uog.70296

**Published:** 2026-08-10

**Authors:** N. Cooper, N. Kotecha, A. Novak, J. Barcroft, J. Yazbek, C. Landolfo, M. Al‐Memar, D. Timmerman, S. Saso, T. Bourne

**Affiliations:** ^1^ West London Cancer Centre, Queen Charlotte's and Chelsea Hospital Imperial College Healthcare NHS Trust London UK; ^2^ Institute for Reproductive and Developmental Biology Imperial College London London UK; ^3^ Gynecology and Obstetrics University Hospitals KU Leuven Leuven Belgium; ^4^ Department of Surgery and Cancer Imperial College London London UK

**Keywords:** diagnostic imaging, magnetic resonance imaging, neoplastic, ovarian cysts, ovarian neoplasms, predictive value of tests, pregnancy, risk assessment, ultrasonography

## Abstract

**Objective:**

To describe the imaging features of adnexal lesions during pregnancy and assess the performance of current models for the risk assessment of such lesions.

**Methods:**

This was a PROSPERO‐registered systematic review including studies published from inception to 1 August 2025 and was performed as per Preferred Reporting Systems for Systematic Reviews and Meta‐Analysis guidance using Covidence software. Studies were included if they utilized an objective or standardized assessment of imaging features (based on the International Ovarian Tumor Analysis (IOTA) terms and definitions, or predefined categorical variables), expert description or quantitative data (such as lesion measurements and number of papillary projections) to assess the risk of adnexal lesions during pregnancy. Standardized extraction of imaging features was performed and classification of outcomes was based on histology, resolution or expert impression. Diagnostic model performance was also extracted. Meta‐analysis of model performance was not performed owing to study heterogeneity.

**Results:**

Overall, 3349 studies were screened, of which 40 underwent full‐text review and 16 were included in the systematic review, comprising 1674 patients and 1660 adnexal lesions. Most included studies were retrospective and ultrasound‐based. Excluding six studies that exclusively reported benign lesions, 93.1% (1284/1379) of patients had benign lesions, 3.4% (47/1379) had borderline ovarian tumors (BOTs) and 3.5% (48/1379) had malignant lesions. Excluding studies reporting exclusively on endometrioma, the most common benign adnexal lesions were simple cysts (39.0% (393/1007)), mature cystic teratomas (24.2% (244/1007)) and endometriomas (14.9% (150/1007)). Malignant lesions were most often epithelial ovarian cancer or metastases. Both BOTs and malignant lesions retained imaging characteristics comparable with those observed in non‐pregnant women. Decidualization was present in 11.8–39.5% of reported endometriomas. While decidualized endometriomas (DEs) and BOTs typically share characteristics, experts noted distinguishing morphological features and behaviors. BOTs were larger (median, 88.0 mm *vs* 48.1 mm), and although both were predominantly unilocular‐solid lesions, BOTs were relatively more often multilocular‐solid (30.0% *vs* 22.4%) compared with DEs. Both showed high vascularity, with most cases having a color score of 3. Most DEs (83.9%) had one to three papillary projections, whereas most BOTs (66.7%) had more than three papillary projections. Diagnostic models, such as the IOTA two‐step strategy and the Assessment of Different NEoplasias in the adneXa model alone, showed high specificity (70.0–90.2%) and negative predictive value (90.0–98.3%), but performance was limited by misclassification of DEs as malignant.

**Conclusions:**

Ultrasound can reliably assess adnexal lesions in pregnancy. DEs and BOTs can appear similar owing to their morphological overlap. Both BOTs and malignant lesions in pregnancy show morphology comparable with that in non‐pregnant women, and IOTA models maintain a high negative predictive value for excluding malignancy in such cases. Future research should refine diagnostic tools to improve the differentiation of benign from malignant cysts with solid components. © 2026 The Author(s). *Ultrasound in Obstetrics & Gynecology* published by John Wiley & Sons Ltd on behalf of International Society of Ultrasound in Obstetrics and Gynecology.

## INTRODUCTION

While adnexal lesions are commonly identified in pregnancy, malignancy is rare in the general obstetric population, with a reported incidence of 1 in 12 000 to 1 in 47 000 pregnancies^1^, ^2^, ^3^, ^4^. A decision to offer intervention is not made lightly, as non‐obstetric surgery during pregnancy is associated with miscarriage, preterm birth and an increased risk of maternal anesthetic complications^5^. Robust preoperative clinical assessment coupled with diagnostic imaging is vital to guide management^6^.

Transvaginal and transabdominal ultrasound are performed safely throughout pregnancy and are recommended as the first‐line imaging modalities in this population^7^, ^8^. Sonographic views may be compromised by the gravid uterus, thus a reliance on transabdominal imaging may be required at more advanced gestation, which can impact the diagnostic value of ultrasound^9^. Cross‐sectional imaging using gadolinium‐based contrast agents is associated with neonatal inflammatory conditions, as well as stillbirth and neonatal death, and so is not recommended during pregnancy^10^. However, diffusion‐weighted magnetic resonance imaging (DW‐MRI) has been shown to improve the diagnostic yield when ultrasound imaging is inconclusive^11^, offering a suitable second‐line investigation.

Ovarian cancer accounts for 3.4% of pregnancy‐associated malignancies^12^. However, it is rarely detected in unselected populations, with an annual detection rate as low as 0%^1^, ^13^. In tertiary units, the prevalence of malignant lesions or borderline ovarian tumors (BOTs) appears to be much higher, with identification in up to 31.9% of pregnant women referred^6^. As with non‐pregnant adult populations, cancers of epithelial origin predominate^6^, ^14^. In expert centers, the accurate diagnosis of adnexal lesions that possess a low risk of progression to invasive cancer, such as BOTs, may enable expectant management with surveillance until the postpartum period^15^.

Pregnancy gives rise to specific diagnostic challenges. The hormonal milieu affects endometriomas and endometriotic nodules, with vascular papillary projections of decidualized endometriomas (DEs) mimicking the appearance of BOTs^16^, ^17^, ^18^, ^19^, and endometriotic deposits presenting as vascular solid pelvic nodules^20^. Current guidance supports fertility‐sparing surgery in the second trimester for BOTs diagnosed early in pregnancy, whereas a DE would require no intervention^21^. Therefore, accurate diagnosis of adnexal lesions forms the basis for subsequent management decisions, which potentially have significant short‐ and long‐term consequences for the mother as well as for the fetus.

Currently, there are no diagnostic models recommended for use in the assessment of adnexal lesions arising in pregnancy^15^. This systematic review aimed to describe the imaging features of adnexal lesions in pregnancy, and to assess the performance of subjective (expert) ultrasound impression and diagnostic tools in discriminating between benign and malignant adnexal lesions.

## METHODS

### Protocol and literature search

This systematic review was conducted in accordance with Preferred Reporting Systems for Systematic Reviews and Meta‐Analysis (PRISMA) guidance^22^. The protocol was registered with PROSPERO (registration no.: CRD42024555115).

A search was performed of the PubMed, EMBASE and The Cochrane Library databases. A combination of the following search terms were used: ‘pregnancy’, ‘ovarian neoplasms’, ‘ovarian cysts’, ‘neoplastic’, ‘ultrasonography’, ‘magnetic resonance imaging’, ‘diagnostic imaging’, ‘risk assessment’ and ‘predictive value of tests’. The full search strategy is available in Appendix S1.

Article titles were screened by two independent reviewers (N.C., N.K.) using Covidence software (Veritas Health Innovation Ltd., Melbourne, Australia), and any disagreement was resolved with a third reviewer (A.N.). Relevant abstracts were then screened independently for inclusion. Reference lists were screened to identify any additional eligible articles for review. J.B., J.Y., C.L., M.A‐.M., S.S., D.T. and T.B. provided input on the clinical interpretation of the extracted findings, from their perspective as imaging experts with expertise in gynecological oncology, fertility, obstetrics, early pregnancy and benign gynecology.

### Eligibility criteria

Original, full‐text research articles published from database inception to 1 August 2025 were included if they utilized an objective or standardized assessment of imaging features (based on the International Ovarian Tumor Analysis (IOTA) terms and definitions, or predefined categorical variables), expert description or quantitative data (such as lesion measurements and number of papillary projections) to assess the risk of adnexal lesions during pregnancy. This was to facilitate a pooling of data to allow objective assessment of imaging features. An ‘expert’ examiner was defined based on the individual study methodology and was self‐reported in each study. Studies were excluded if they lacked imaging descriptions, were case reports or case series involving fewer than five patients, were not written in English and did not have an English translation, evaluated risk assessment tools for adnexal lesions solely outside of pregnancy, or did not adhere to current accepted ethical standards (Appendix S2).

### Data extraction

Data were extracted from each study according to predefined criteria. Study characteristics, including country, study design, type of center, inclusion and exclusion criteria and study duration, were recorded. Participant data, including sample size, number of adnexal lesions, patient age and number of benign, borderline and malignant lesions, were also extracted. For ultrasound‐based studies, standardized data extraction of imaging features was performed to capture lesion appearances using the IOTA terms and definitions^23^, as well as the results of quantitative risk‐assessment tools. Studies were not excluded if they did not use the IOTA terms and definitions. The MRI features that were extracted were descriptive and quantitative morphological features (e.g. lesion size, solid components, septations), signal characteristics on T1‐ and T2‐weighted MRI and the presence or absence of contrast enhancement, when applicable.

### Quality assessment

The risk of bias was assessed independently by one author (N.K.). The Downs and Black^24^ critical appraisal checklist was used to determine the overall quality of the included studies. This tool has been widely applied in studies with comparable designs. The checklist comprises 26 items grouped into four categories: reporting, external validity, internal validity and confounding. Each study was reviewed using this framework, with responses recorded as ‘yes’, ‘no’ or ‘unclear/not applicable’, depending on the availability of relevant information. A total score was then calculated. Studies scoring below 14 were considered to have methodological weaknesses, scores between 15 and 19 indicated moderate quality, scores of 20–24 represented good quality and scores above 25 reflected excellent methodological rigor.

### Data synthesis

Given the descriptive nature of this systematic review, we used descriptive statistics to report demographic and clinical characteristics, reported as mean ± SD, median (interquartile range) or median (range) for continuous variables and *n* (%) for categorical variables. Test performances are reported using sensitivity, specificity, positive predictive value (PPV), negative predictive value (NPV) and/or the area under the receiver‐operating‐characteristics curve, depending on what was provided in each study. Pooled analyses of the objective ultrasound characteristics of adnexal lesions were undertaken using IOTA terminology, when presented in the original study. If it was unclear whether IOTA terms and definitions were used to describe a characteristic, these data were marked as ‘not available’. Outcome‐based analyses were restricted to adnexal lesions with a confirmed final diagnosis, classified as malignant or borderline based on histology, or as benign based on histology, documented follow‐up or spontaneous resolution.

To minimize the potential risk of duplication bias due to overlapping populations in studies conducted by the same authors or at the same institution, we contacted the corresponding authors of the articles following data extraction. In cases in which studies included the same population, the study with the smaller sample size or the older publication was excluded, or if the newer publication had a smaller sample size, the publication with the larger sample was prioritized. Additionally, to calculate the prevalence of benign, borderline and malignant lesions, a subanalysis was conducted in which studies restricted exclusively to benign or exclusively to malignant lesions were excluded from the primary descriptive analysis.

## RESULTS

### Clinical and study characteristics

Overall, 3362 titles were identified, and after removal of duplicates, 3349 titles were screened, and 40 full‐text articles were assessed for eligibility (Figure 1). In total, 16 studies were included in the systematic review^1^, ^2^, ^6^, ^9^, ^17^, ^18^, ^19^, ^20^, ^25^, ^26^, ^27^, ^28^, ^29^, ^30^, ^31^, ^32^, including 1674 patients and 1660 adnexal lesions (Tables 1 and 2). Fewer lesions were identified than patients, as one study included some patients with non‐ovarian endometriosis^20^ and one study excluded patients with clearly benign lesions who did not require referral to a gynecologist–oncologist for counseling^30^. The greatest proportion of the studies (5/16 (31.3%)) were conducted in joint gynecology–oncology and early pregnancy centers. Six (37.5%) studies were prospective, and the remainder (10/16 (62.5%)) were retrospective. The number of participants in each study ranged from nine to 291, with malignant lesions identified in 0–25.0% of unselected cohorts. Of the studies presenting patient age as a median (*n* = 12), the mean age was 31.8 (range, 17–47) years (Table 2).

**Figure 1:**
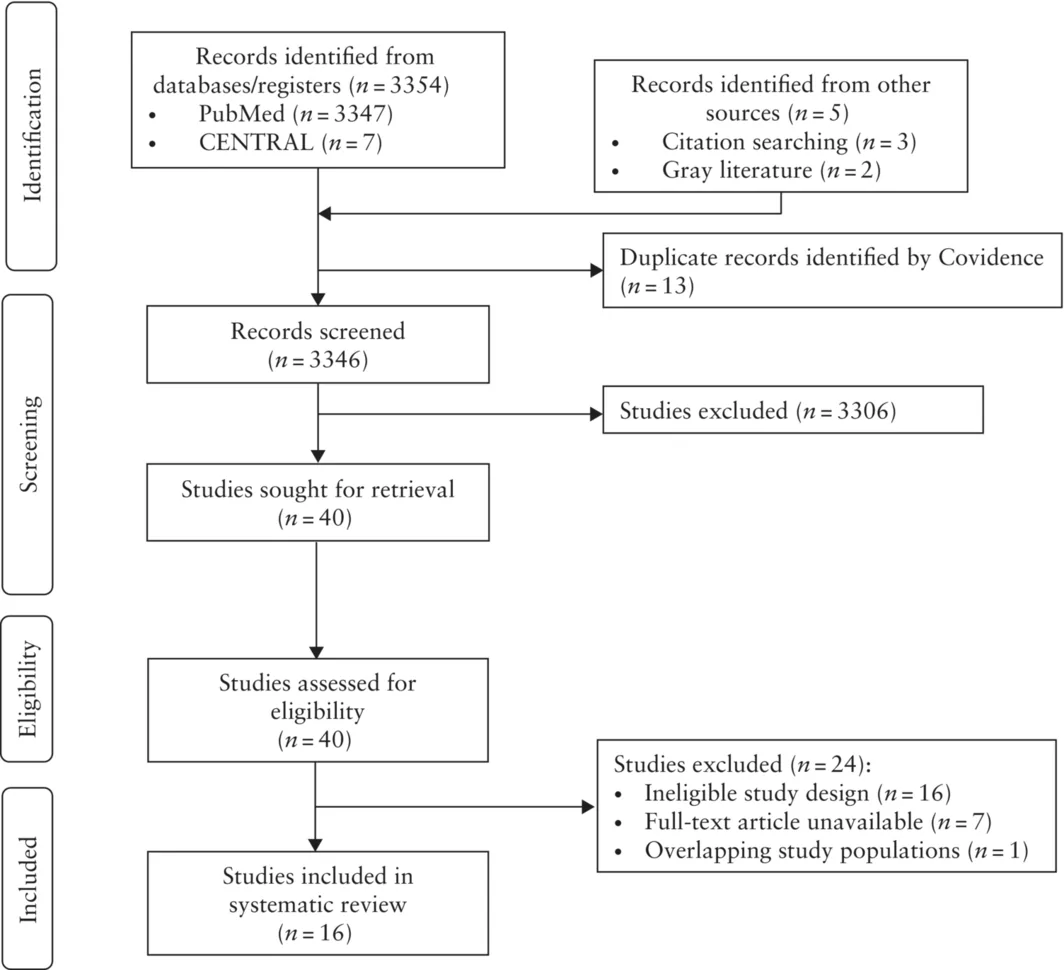
Flowchart showing inclusion in systematic review of studies assessing the imaging features of adnexal lesions during pregnancy.

**Table 1:** Characteristics of 14 studies included in systematic review on imaging features of adnexal lesions in pregnancy

Study	Country	Study design	Study period	Type of center	Inclusion criteria	Exclusion criteria
Barcroft (2024)^9^	UK	Retro analysis of prospectively recruited patients	March 2017–August 2022	Gynecology–oncology center and early pregnancy unit	Pregnant women aged ≥ 18 years with a non‐physiological adnexal lesion ≥ 1 cm on US	Patient age < 18 years
Bean (2023)^20^	UK	Prosp observational cohort	September 2017–September 2020	Gynecology–oncology center and early pregnancy unit	Pregnant women aged 18–50 years with an endometrioma or deep endometriosis identified in early pregnancy	NS
Bromley (1997)^25^	USA	Retro cohort	1988–1995	University hospital	Pregnant women with an ovarian lesion > 4 cm in size and persisting beyond 12 weeks' gestation	NS
Condous (2004)^1^	UK	Prosp observational longitudinal	June 2001–July 2002	Gynecology–oncology center and early pregnancy unit	Consecutive pregnant women attending an early pregnancy unit diagnosed with an ovarian lesion	Simple cyst with maximum diameter < 25 mm
Czekierdowski (2021)^26^	Poland	Retro cohort, multicenter	September 2013 – December 2020	Multicenter (*n* = 2)	Pregnant women with a surgically managed ovarian mass and antenatal ultrasound images	Simple cyst or CL cyst
Gaughran (2024)^2^	UK	Prosp observational cohort	January 2019–August 2021	Gynecology–oncology center and early pregnancy unit	Consecutive pregnant women attending an early pregnancy unit or routine prenatal US scan diagnosed with an ovarian lesion	History of ovarian BOT or malignancy, CL cyst with maximum diameter < 30 mm
Groszmann (2014)^27^	USA	Retro cohort	January 2005–December 2012	Women's hospital	Women with a histological diagnosis of decidualized endometrioma who had undergone preoperative US in pregnancy	NS
Lee (2021)^28^	South Korea	Retro cohort, multicenter	January 2005–December 2017	Multicenter (*n* = 11)	Women with a histological diagnosis of a lesion operated on during or within 3 months of pregnancy who had undergone preoperative US in pregnancy	NS
Mascilini (2014)^17^	Italy	Retro cross‐sectional observational	2003–2011	Multicenter (*n* = 7)	Women with a histological diagnosis of decidualized endometrioma who had undergone a preoperative US in pregnancy	NS
Orlov (2025)^18^	Sweden	Prosp observational cohort	2013–2024	Tertiary referral center	Pregnant women diagnosed with ovarian endometrioma during pregnancy	NS
Panico (2023)^29^	Italy	Retro cohort	January 2011–February 2023	Tertiary referral center	Pregnant women diagnosed with an ovarian mass with an indeterminate US diagnosis and who underwent MRI in pregnancy	Extraovarian mass
Pateman (2014)^19^	UK	Retro cohort	January 2009–May 2013	Gynecology–oncology center and early pregnancy unit	Pregnant women diagnosed with an ovarian endometrioma at an early pregnancy unit	NS
Rabiej‐Wronska (2022)^30^	Poland	Prosp observational cohort	January 2015–December 2017	Gynecology–oncology center	Consecutive pregnant women referred for ultrasonographic assessment of an adnexal lesion	NS
Takeuchi (2016)^31^	Japan	Retro case–control	March 2005–July 2013	University hospital	Women with a decidualized endometrioma who underwent MRI in pregnancy, compared to non‐pregnant women with confirmed ovarian cancer	NS
Testa (2021)^6^	Italy	Retro cohort	December 2019–January 2000	Gynecology–oncology center	Pregnant women diagnosed with an ovarian lesion during pregnancy	NS
Zanetta (2003)^32^	Italy	Prosp observational cohort	January 1996–December 1999	Women's hospital	Consecutive pregnant women attending a maternity unit diagnosed with an ovarian lesion	CL cyst with maximum diameter < 30 mm

Only first author is given for each study. BOT, borderline ovarian tumor; CL, corpus luteum; MRI, magnetic resonance imaging; NS, none stated; prosp, prospective; retro, retrospective; US, ultrasound.

**Table 2:** Characteristics of pregnant patients with adnexal lesion included in systematic review

				Lesion type			
Study	Patients (*n*)	Lesions (*n*)	Age (years)	Benign	Borderline	Malignant	N/A
Barcroft (2024)^9^	291	291	33 (29–36)	256 (88.0)	11 (3.8)	0 (0)	24 (8.2)
Bean (2023)^20^, *	65	34	34 [23–44]	34 (100)	0 (0)	0 (0)	0 (0)
Bromley (1997)^25^	125	131	[21–47]	130 (99.2)	0 (0)	1 (0.8)	0 (0)
Condous (2004)^1^	161	166	30 (17–32)	165 (99.4)	1 (0.6)	0 (0)	0 (0)
Czekierdowski (2021)^26^	36	36	28.5 (20–42)	27 (75)	2 (5.6)	7 (19.4)	0 (0)
Gaughran (2024)^2^, †	274	282	32.03 (19–45)	272 (99.3)	2 (0.7)	0 (0)	0 (0)
Groszmann (2014)^27^	17	22	29 [22–43]	22 (100)	0 (0)	0 (0)	0 (0)
Lee (2021)^28^	236	236	31.7 ± 4.2 (benign); 33.5 ± 5.0 (malignant)	223 (94.5)	0 (0)	13 (5.5)	0 (0)
Mascilini (2014)^17^	18	18	34 [20–43]	18 (100)	0 (0)	0 (0)	0 (0)
Orlov (2025)^18^	57	57	31 (29–34)	57 (100)	0 (0)	0 (0)	0 (0)
Panico (2023)^29^	20	20	31.3 (20–41)	13 (65.0)	0 (0)	5 (25.0)	2 (10.0)
Pateman (2014)^19^, ‡	24	34	35 (32–37)	34 (100)	0 (0)	0 (0)	0 (0)
Rabiej‐Wronska (2022)^30^, §	153	134	28.0 ± 5.48 (benign); 28.5 ± 6.09 (malignant)	49 (36.6)	6 (4.5)	6 (4.5)	73 (54.5)
Takeuchi (2016)^31^	9	9	31 (25–37)	9 (100)	0 (0)	0 (0)	0 (0)
Testa (2021)^6^	113	113	33 (19–44)	77 (68.1)	20 (17.7)	16 (14.2)	0 (0)
Zanetta (2003)^32^	75	77	—	72 (93.5)	5 (6.5)	0 (0)	0 (0)

Only first author is given for each study. Data are given as *n*, median (interquartile range), median [range], [range], mean ± SD or *n* (%).

* Sum of patients is greater than sum of lesions as 31 patients with non‐ovarian endometriosis were included.

† Eight lesions were bilateral; histology data are presented for total number of patients.

‡ 10 patients had > 1 lesion; histology data are presented for total number of lesions.

§ Sum of patients is greater than sum of lesions as 19 patients with clearly benign lesions who did not require referral to a gynecologist–oncologist were included. N/A, not available.

Overall, 969/1640 (59.1%) cases were initially managed conservatively, of which 439/969 (45.3%) lesions resolved prenatally, and 648/1640 (39.5%) patients underwent surgery. Of these, 445 (68.7%) underwent surgery during pregnancy. In total, 193/445 (43.4%) had surgery at the time of Cesarean section (Table S1).

Tumor histological subtypes are described in Figure S1 and Table S2. Excluding six studies with selected populations (in which exclusively benign lesions were included)^17^, ^18^, ^19^, ^20^, ^27^, ^31^, there were 1379 lesions for which the final histological classification was available (benign, borderline or malignant), of which 93.1% (*n* = 1284) were benign lesions, 3.4% (*n* = 47) were BOTs and 3.5% (*n* = 48) were malignant lesions (Table 2).

### Lesion characteristics on imaging

Table S3 outlines the imaging features of adnexal lesions arising in pregnancy. Analyses of histological subtype were restricted to the subset of lesions for which a specific subtype was reported. Thirteen studies assessed ultrasound findings and reported proposed and/or histologically confirmed tumor subtypes (Tables S2, S4–S6).

#### Benign adnexal lesions

Of the 1414 benign lesions assessed across the 13 studies, a subtype was documented for 1159; the remaining 255 lesions were reported as benign but not assigned a subtype. Of these benign lesions, the most common were simple cysts (33.9% (393/1159)), endometriomas (26.1% (302/1159)) and mature cystic teratomas (21.1% (244/1159)). Histology data were not reported in 233 benign lesions and subtype was specified as unavailable for 24 benign lesions. Of the 302 endometriomas, 152 (50.3%) were from selected populations (in which exclusively endometriomas were included)^17^, ^18^, ^19^, ^20^, ^31^, of which one study assessed their appearance on MRI only (5.9% (9/152))^31^. Excluding these cases, there were 1007 benign lesions with subtype data available, of which 39.0% (*n* = 393) were simple cysts, 14.9% (*n* = 150) were endometriomas and 24.2% (*n* = 244) were mature cystic teratomas.

The imaging features of benign tumors are outlined in Tables S3 and S4. Of 399/451 benign lesions with available diameter data, the median diameter was 71 (range, 15–266) mm (Table S4). The extent to which lesions were described using IOTA terminology varied. Most benign lesions were unilocular (48.9% (109/223)) with anechoic content (39.1% (117/299)) and a color score of 1 (61.4% (94/153)). Solid papillary projections were present in 17.2% (60/349) of benign lesions and 41.7% (25/60) of these projections showed Doppler color flow within them.

#### Endometriomas

Six studies provided morphological data on 206 endometriomas, summarized in Tables S3 and S5, ^9^, ^17^, ^18^, ^19^, ^20^, ^27^. Two studies reported exclusively DEs^17^, ^27^, while the remainder included all endometriomas. Of the studies not reporting exclusively DEs, decidualization occurred in 11.8–39.5% of endometriomas.

DEs were described as vascular, with rounded papillary projections and irregular walls. Three studies described regression of the size of the lesion throughout gestation^18^, ^19^, ^20^, and one described an increase in the size of the solid components^18^. Postpartum resolution was a key factor in establishing the benign nature of a lesion. There was one case of a mistaken BOT among 111 (0.9%) DEs during pregnancy, however this lesion was subsequently recognized as a BOT at the postpartum scan^9^.

Quantitative morphological data were available for DEs in three studies (*n* = 58 lesions)^9^, ^17^, ^27^. The median maximum diameter was 48.1 (range, 11.3–121.0) mm. Most DE lesions were unilocular‐solid (53.4% (31/58)) or multilocular‐solid (22.4% (13/58)). Most had a single papillary projection (45.2% (14/31)) and 84.4% (38/45) of papillary projections had vascular flow. Most had ground‐glass (52.6% (20/38)) or low‐level content (28.9% (11/38)) and a color score of 3 (39.7% (23/58)).

#### Borderline and malignant adnexal lesions

Tables 3, S3 and S6 outline the imaging features of BOTs and malignant lesions. Both BOTs and malignant lesions retained imaging characteristics seen in non‐pregnant women. Histological subtypes were described for 41 BOTs. Serous BOTs were the most common (61.0% (25/41)), followed by mucinous BOTs (29.3% (12/41)). Malignant histologies were described for 92.7% (38/41) and described as ‘other’ for 7.3% (3/41). Epithelial ovarian cancer (EOC) (57.9% (22/38)) and metastases (21.1% (8/38)) were the most common malignant adnexal lesions identified. Most EOCs were serous adenocarcinomas (45.5% (10/22)) followed by mucinous adenocarcinomas (27.3% (6/22)) and endometrioid adenocarcinomas (18.2% (4/22)).

**Table 3:** Imaging features, described using Internation Ovarian Tumor Association (IOTA) terms and definitions, of benign lesions, decidualized endometriomas (DEs), borderline ovarian tumors (BOTs) and malignancies, based on pooled data from included studies

Characteristic	Benign (*n* = 451)	DE (*n* = 111)	BOT (*n* = 20)*	Malignancy (*n* = 29)†
Lesion diameter (mm)	71 (15–266)‡	48.1 (11.3–121.0)	88.0 (37–216)	115.4 (43–182)
Lesion type				
Unilocular	109/223 (48.9)	6/58 (10.3)	0 (0)	0/26 (0)
Unilocular‐solid	54/223 (24.2)	31/58 (53.4)	9 (45.0)	2/26 (7.7)
Multilocular	34/223 (15.2)	5/58 (8.6)	4 (20.0)	0/26 (0)
Multilocular‐solid	15/223 (6.7)	13/58 (22.4)	6 (30.0)	8/26 (30.8)
Solid	11/223 (4.9)	3/58 (5.2)	1 (5.0)	16/26 (61.5)
Content				
Anechoic	117/299 (39.1)	7/38 (18.4)	—	2/6 (33.3)
Low‐level	70/299 (23.4)	11/38 (28.9)	—	4/6 (66.7)
Ground‐glass	74/299 (24.7)	20/38 (52.6)	—	0/6 (0)
Hemorrhagic	5/299 (1.7)	0/38 (0)	—	0/6 (0)
Mixed	33/299 (11.0)	0/38 (0)	—	0/6 (0)
Color score				
1	94/153 (61.4)	13/58 (22.4)	4 (20.0)	0/16 (0)
2	31/153 (20.3)	13/58 (22.4)	7 (35.0)	3/16 (18.8)
3	24/153 (15.7)	23/58 (39.7)	9 (45.0)	8/16 (50.0)
4	4/153 (2.6)	8/58 (13.8)	0 (0)	5/16 (31.3)
Solid components				
Presence of solid papillary projections	60/349 (17.2)	45/58 (77.6)	15 (75.0)	8/29 (27.6)
Presence of color flow in papillary projections	25/60 (41.7)	38/45 (84.4)	15/15 (100.0)	6/6 (100)
Stage				
Ia	—	—	—	2/11 (18.2)
Ib	—	—	—	0/11 (0)
Ic	—	—	—	4/11 (36.4)
IIb	—	—	—	1/11 (9.1)
IIIa	—	—	—	0/11 (0)
IIIa	—	—	—	0/11 (0)
IIIc	—	—	—	3/11 (27.3)
IV	—	—	—	1/11 (9.1)

Data are given as median (range), *n/N* (%) or *n* (%).

* Only including cases from Testa (2021)^6^.

† Only including cases from Lee (2021)^28^ and Testa (2021)^6^ (mixed BOT and malignant datasets in Rabiej‐Wronska (2022)^30^ and Czekierdowski (2021)^26^ are excluded).

‡ Diameter data available for 399 lesions.

Imaging features were described in only one study, for 20 BOTs^6^. The median lesion diameter was 88.0 (range, 37–216) mm. Most BOTs were unilocular‐solid (45.0% (*n* = 9)), multilocular‐solid (30.0% (*n* = 6)) or multilocular (20.0% (*n* = 4)). Of the multilocular and multilocular‐solid BOTs (*n* = 10), most (60% (*n* = 6)) had ≤ 10 cyst locules. Most BOTs had a color score of 2 or 3 (35.0% (*n* = 7) and 45.0% (*n* = 9), respectively). The majority of BOTs had at least one solid papillary projection present (75.0% (15/20)) and most lesions had more than three solid papillary projections (66.7% (10/15)). All papillary projections were vascular.

Tumor features were described for 29 malignancies. The median diameter of malignant lesions was 115.4 (range, 43–182) mm. Most malignant lesions were multilocular‐solid (30.8% (8/26)) or solid (61.5% (16/26)). Of the multilocular and multilocular‐solid lesions, most had ≤ 10 cyst locules (76.2% (16/21)). Cyst content was described for six lesions, of which most had low‐level content (66.7% (4/6)). Color score was described in 16 lesions, of which 50% had a color score of 3. Solid papillary projections were identified in 27.6% (*n* = 8) of lesions. The number of papillary projections was described in six cases; most had more than three solid papillary projection (5/6 (83.3%)), all of which had vascular flow. Ascites was present in one case (1/13 (7.7%)). The International Federation of Gynecology and Obstetrics (FIGO) stage was reported for 11 cases of malignancy: most patients presented with Stage‐I (6/11 (54.5%)) or Stage‐III (3/11 (27.3%)) disease.

### Comparison of decidualized endometriomas and borderline ovarian tumors

BOTs tended to be slightly larger than DEs on average (median, 88.0 mm *vs* 48.1 mm) (Table 3). Based on pooled BOT and malignancy data, BOTs had mostly low‐level content compared with DEs, which more often had ground‐glass content. Both were predominantly unilocular‐solid (BOTs, 45.0% *vs* DEs, 53.4%), but BOTs were relatively more often multilocular‐solid (30.0% *vs* 22.4%). For both lesion types, most cases were described as highly vascular with a color score of 3. However, 83.9% of DEs had one to three papillary projections, while 66.7% of BOTs had more than three papillary projections. Both BOTs and DEs had vascular flow within papillary projections in most cases (100% *vs* 84.4%).

One study assessed the role of DW‐MRI in the differentiation of DEs from BOTs^31^. DEs demonstrated different signal intensities on diffusion‐weighted imaging (DWI)‐800 compared with the high signal intensity observed on both DWI‐1500 and DWI‐800 in malignant ovarian tumors in a control group of non‐pregnant women. Higher b‐value DWI sequences increase sensitivity to diffusion restriction, which is commonly seen in malignant lesions.

### Magnetic resonance imaging findings

Two studies assessed MRI findings. Takeuchi *et al*.^31^ described five DEs as unilocular cysts with T2‐hyperintense mural papillary projections of variable morphology, with lesion sizes ranging from 4 cm to 9 cm and diagnosis occurring between 9 and 20 weeks' gestation. Cyst contents were T1‐hyperintense, consistent with hemorrhagic material. When DW‐MRI was performed, decidualized mural nodules demonstrated higher apparent diffusion coefficient (ADC) values compared with malignant mural nodules. However, the comparison group comprised non‐pregnant women with ovarian cancer. Panico *et al*.^29^ found that non‐contrast MRI could be used to identify malignancy and use of the non‐contrast MRI score (NCMS) enhanced identification of cancers by non‐expert radiologists. They used DWI b‐values of 0 and 800–1000 s/mm^2^ and similarly found significantly higher ADC values in benign lesions compared with malignancies.

### Performance of diagnostic models

Six studies assessed diagnostic models (Tables 4 and S7)^2^, ^9^, ^26^, ^28^, ^29^, ^30^. Meta‐analysis of these diagnostic models was not feasible given the heterogeneity in the approaches used.

**Table 4:** Summary of diagnostic models

Model	*n*	Sensitivity (%) (95% CI)	Specificity (%) (95% CI)	PPV (95% CI)	NPV (95% CI)	AUC	Source
IOTA Simple Rules							
Cut‐off of 1*	61	100 (73.5–100)	51.0 (36.3–65.6)	33.3 (27.3–40.0)	100	N/A	Rabiej‐Wronska (2022)^30^
Cut‐off of 3	61	91.7 (61.5–99.8)	53.1 (38.3–67.5)	32.4 (25.3–40.1)	96.3 (79.6–99.4)	N/A	Rabiej‐Wronska (2022)^30^
Cut‐off of 30	61	91.7 (61.6–99.8)	73.5 (58.9–85.1)	45.8 (34.0–58.2)	97.3 (84.6–99.6)	N/A	Rabiej‐Wronska (2022)^30^
Exclusion of inconclusive results	61	87.5 (47.4–99.7)	94.4 (81.3–99.3)	77.8 (47.0–93.2)	97.1 (84.4–99.5)	N/A	Rabiej‐Wronska (2022)^30^
Classification of inconclusive results as probably malignant	61	91.7 (61.5–99.8)	69.4 (54.6–81.8)	42.3 (31.8–53.6)	97.1 (83.8–99.6)	N/A	Rabiej‐Wronska (2022)^30^
Simple Rules Risk*	36	100	37.0	35.0	100	N/A	Czekierdowski (2021)^26^
Simple Rules†	274	N/A	N/A	N/A	N/A	N/A	Gaughran (2024)^2^
IOTA ADNEX							
2‐step strategy	83	63.6 (35.2–92.1)	90.2 (86.6–93.9)	21.9 (7.6–36.2)	98.3 (96.6–100)	N/A	Barcroft (2024)^9^
Cut‐off > 20%*	36	78.0	70.0	47.0	90.0	N/A	Czekierdowski (2021)^26^
Cut‐off > 10%*	236	61.5 (31.6–86.1)	84.8 (79.4–89.2)	19.0	97.4	0.709	Lee (2021)^28^
ROMA*	24	25.0	100.0	100.0	73.0	0.75	Czekierdowski (2021)^26^
Sassone*	236	69.2 (38.6–90.9)	84.8 (79.4–89.2)	20.9	97.9	0.831	Lee (2021)^28^
Lerner*	236	76.9 (46.2–95.0)	68.6 (62.1–74.6)	12.5	98.1	0.710	Lee (2021)^28^
Combined model*	236	69.2 (38.6–90.9)	94.6 (90.8–97.2)	42.9	98.1	0.883	Lee (2021)^28^
Non‐contrast MRI score	20	85.7 (59.8–100)	76.9 (54.0–99.8)	66.7 (35.9–97.5)	90.9 (73.9–100)	N/A	Panico (2023)^29^
IOTA Benign Descriptors‡	83	N/A	N/A	N/A	N/A	N/A	Barcroft (2024)^9^

Only first author is given for each study.

* 95% CI not available for all or partial data.

† International Ovarian Tumor Association (IOTA) Simple Rules applicable in 272/274 (99.3%) lesions.

‡ IOTA Benign Descriptors applicable in 184/267 (68.9%) lesions, and risk of malignancy was < 0.5%, where applicable. ADNEX, Assessment of Different NEoplasias in the adneXa; AUC, area under the receiver‐operating‐characteristics curve; MRI, magnetic resonance imaging; N/A, not available; NPV, negative predictive value; PPV, positive predictive value; ROMA, Risk of Ovarian Malignancy Algorithm.

Barcroft *et al*.^9^ assessed the IOTA two‐step strategy (IOTA benign descriptors (BDs) followed by the Assessment of Different NEoplasias in the adneXa (ADNEX) model) in 83 patients, in which it performed with a specificity of 90.2% (95% CI, 86.6–93.9%) and a NPV of 98.3% (95% CI, 96.6–100%). Sensitivity was 63.6% (95% CI, 35.2–92.1%) with a PPV of 21.9% (95% CI, 7.6–36.2%), attributed to the misclassification of DEs as malignant. When assessing the BDs alone, they were applicable in 68.9% of the population and, where applicable, the risk of malignancy was < 0.5%. Two further studies assessed the ADNEX model^26^, ^28^. Czekierdowski *et al*.^26^ found that the ADNEX model using a higher cut‐off of > 20% performed with a lower sensitivity and specificity of 78.0% and 70.0%, respectively. The study of Lee *et al*.^28^ also assessed the ADNEX model, in which it performed similarly, with a specificity of 84.8%, NPV of 97.4%, sensitivity of 61.5% and PPV of 19.0%. This study also assessed the Sassone and Lener scoring systems, achieving similar diagnostic performance.

Gaughran *et al*.^2^ assessed the IOTA Simple Rules, which were applicable to 99.3% of lesions, but the diagnostic performance was not calculated. The IOTA Simple Rules were also assessed in the study of Rabiej‐Wronska *et al*.^30^ at various cut‐offs, performing with a specificity of 94.4% and a NPV of 97.1% when the model was used as intended (with inconclusive results excluded and flagged for further assessment).

### Risk of bias

A risk‐of‐bias score was allocated for each study (Figure S2). Overall, 9/16 (56.3%) studies scored 15–19, indicating evidence of moderate quality, and 5/16 (31.3%) studies scored below 15, indicating a degree of bias in the study design. The remaining two (12.5%) studies scored 20 or above, indicating good‐quality evidence.

## DISCUSSION

This systematic review demonstrates that most adnexal lesions arising in pregnancy are benign, that ultrasound‐based assessment, including IOTA‐derived diagnostic tools, shows a high NPV for malignancy, and that DEs remain the principal diagnostic challenge in cases of adnexal lesions because of their morphological overlap with BOTs.

In the present study, almost half of conservatively managed lesions resolved prenatally, suggesting a predominance of functional ovarian cysts. In the published literature, such spontaneously resolving lesions have typically been reported to resolve prior to 20 weeks and to be unilocular, with mostly anechoic content and a color score of 1^1^, ^2^. This is consistent with the observational clinical study of Yazbek *et al*.^13^, in which 84.8% of simple cysts ≥ 5 cm and ‘complex’ cysts resolved during pregnancy. Therefore, such cysts should generally be managed conservatively.

Benign lesions were most often simple cysts, endometriomas or mature cystic teratomas, all of which can be classified using the IOTA‐BDs if they measure < 10 cm in size^18^, ^19^, ^20^. The diagnostic study of Barcroft *et al*.^9^ found that the IOTA‐BDs remained applicable in 68.9% of adnexal masses in the pregnant population investigated, with a risk of malignancy of < 0.5% when a BD is applicable. When lesions do not resolve, pregnant women can be reassured that most adnexal cysts are benign and most can be safely managed without intervention. Surgical management may be considered for a benign lesion if the patient is symptomatic, including approaches such as percutaneous drainage, laparoscopy, laparotomy and opportunistic removal during Cesarean section.

Ovarian malignancy is rare in low‐risk populations. In their study, Yazbek *et al*.^13^ found no cases of malignancy among 2925 consecutive women undergoing a routine 11–14‐week obstetric scan. Therefore, the prevalence of malignancy is likely to be overestimated in the included studies owing to a predominance of high‐risk referral centers.

Malignant ovarian tumors were rare, comprising just 3.5% (48/1379) of all lesions in our cohort, and all received surgical intervention either during pregnancy or postpartum. Most malignancies were early stage, but a proportion presented with Stage‐III disease^33^. Histological subtypes were consistent with those of non‐pregnant adult women, with a predominance of primary EOC and metastases. ESGO/INCIP (European Society of Gynecological Oncology/International Network on Cancer, Infertility and Pregnancy) recommend different surgical management approaches depending on gestational age, apparent stage of disease and histological subtype. Early‐stage disease may be managed surgically at any gestation, however the pouch of Douglas is usually inaccessible beyond 18 weeks, therefore restaging after delivery would be required. Biopsy and non‐surgical management of advanced EOC in pregnancy are currently recommended beyond 18 weeks, alongside neoadjuvant chemotherapy and postpartum debulking. Prior to 18 weeks, cytoreductive surgery with termination of pregnancy can be considered; however, if a patient wishes to continue the pregnancy, a similar management strategy to that for EOC arising at later gestation may still be adopted^21^.

The morphological features of malignancies were consistent with those in non‐pregnant women: malignant lesions were larger than benign lesions and BOTs, with multilocular‐solid or solid morphology and significant vascularity. Therefore, the presence of such features should immediately warrant referral to an expert gynecology–oncology center, with cross‐sectional assessment using DW‐MRI recommended as per expert consensus^21^.

Decidualization is common and was observed in 11.8–39.5% of endometriomas^9^, ^17^, ^18^, ^19^, ^20^, ^27^. There was only one case of a BOT misdiagnosed as a DE during pregnancy, but this was identified at the postpartum scan^9^. Based on morphology alone, the characteristics of BOTs *vs* those of DEs are difficult to differentiate; however, DEs have been widely described as having smooth and rounded papillary projections, in contrast to the craggy or ‘cauliflower‐like’ projections seen in BOTs^6^, ^14^, ^17^, ^18^, ^19^, ^20^. Longitudinal assessment also aids evaluation, with serial expert assessment allowing reassurance regarding the nature of a lesion. The reported ‘expected’ behavior of a DE during pregnancy was described as regression in size with advancing gestation, increase in size of solid components beyond 22 weeks and subsequent resolution of solid components either in the third trimester or postpartum^2^, ^9^, ^17^, ^18^, ^19^. Ultrasound evidence of a typical endometrioma identified prior to pregnancy is particularly reassuring, as well as other features of endometriosis such as nodules or reduced mobility of the pelvic organs^18^, ^20^.

Based on the literature and our experience in a dedicated ‘cyst in pregnancy’ clinic, we suggest that all women with a newly suspected DE are referred for expert review within 2 weeks. A focused patient history review should establish any symptoms or known diagnosis of endometriosis, and antecedent imaging should be reviewed. Transvaginal ultrasound should be utilized as the first‐line examination, as endometriomas typically remain visible vaginally even in advanced pregnancy owing to their frequent adherence to the posterior aspect of the uterus. Assessment should include additional sonographic markers of endometriosis in accordance with the International Deep Endometriosis Analysis consensus^34^. Reassuring morphological features include smooth or rounded papillary projections, a maximum diameter of < 10 cm, ground‐glass content and fewer than four papillary projections. Vascularity is generally less discriminative. We suggest longitudinal surveillance every 4–6 weeks from 12 to 34 weeks' gestation, with postpartum review 6 weeks after delivery to confirm resolution.

From our experience, differentiating between clear‐cell carcinoma and decidualization can be particularly challenging. There were two cases of clear‐cell carcinoma in the included studies, but none described their characteristics specifically. Pozzati *et al*.^35^ describe clear‐cell carcinoma in non‐pregnant adults as a unilateral unilocular‐solid or multilocular‐solid lesion, often with ground‐glass content and vascular papillary projections, with a median diameter of 117 mm. There are insufficient data to decide whether clear‐cell carcinomas are affected by pregnancy, however the described morphology of such lesions in non‐pregnant women is similar to the characteristics of DEs and BOTs. Again, larger lesion diameter, longitudinal assessment and lack of regression would be key indicators of malignancy.

BOTs tend to grow slowly, and the risk of progression to an invasive carcinoma is as low as 2% in non‐pregnant women^36^. Based on our experience in a high‐risk oncology center, we have not observed any cases of a BOT progressing to an invasive cancer when managed expectantly in pregnancy. Therefore, conservative management followed by postpartum surgery is theoretically also feasible in this population, if one can be confident that there is no invasive disease present^37^.

There are inadequate data to assess the diagnostic superiority of MRI for adnexal lesions in pregnancy, and it is not routinely recommended as a first‐line imaging modality. DW‐MRI has been recommended for radiological staging if invasive disease is suspected, as well as in the case of an inconclusive ultrasound examination^21^. Panico *et al*.^29^ found that the NCMS can be used by non‐expert radiologists, and improved the classification of benign and malignant lesions. The study of Takeuchi *et al*.^31^ also suggests that the signal intensity pattern may differ in DEs compared with in malignant lesions, but it did not compare DEs with BOTs in pregnancy.

The IOTA Simple Rules, IOTA two‐step strategy and IOTA ADNEX model alone each performed with a high NPV and specificity, which reinforces that benign lesions can be confidently risk assessed in this population. However, misclassification of DEs as BOTs contributes to false‐positive results, limiting clinical applicability in pregnancy. Based on our findings, no model can be recommended for use in pregnancy, however it is likely that the IOTA‐BDs remain applicable. The IOTA in Pregnancy study aims to prospectively assess the performance of the IOTA two‐step strategy, with results expected in the next 3 years^38^. At present, the combination of expert assessment and longitudinal observation is the best management strategy for these patients, with evidence that the IOTA‐BDs retain their performance where applicable.

This systematic review includes predominantly ultrasound‐based studies, limiting its ability to assess the diagnostic accuracy of MRI for adnexal lesions. Furthermore, the small number of malignant cases limits the statistical power to meaningfully differentiate between BOT, DE and clear‐cell carcinoma. Additionally, many of the included centers were tertiary gynecology–oncology centers, inflating the prevalence of malignant disease. Furthermore, studies describing the behavior of endometriosis in pregnancy introduced selection bias. The six studies evaluating diagnostic models were also heterogeneous; four were retrospective and four were single‐center studies, thus limiting the generalizability of their findings and precluding a meta‐analysis of model performance. Risk‐of‐bias assessment described most evidence as moderate or poor quality overall.

Clinically, these findings support the conservative management of benign‐appearing adnexal lesions. Most benign lesions retain the same imaging features as those observed outside pregnancy. Similarly, cancers demonstrate the same typical imaging features in pregnant and non‐pregnant women and should prompt urgent referral to a tertiary gynecology–oncology center. In cases in which there is diagnostic uncertainty, expert ultrasound assessment with longitudinal evaluation may be a useful tool until accurate diagnostic tools have been developed. The role of other imaging modalities, such as MRI, requires further prospective validation studies. There may also be a potential role of artificial intelligence (AI) in the differentiation of solid components identified in BOTs and DEs, as both AI and radiomics have been shown to provide better risk stratification of adnexal lesions^39^, ^40^. As such, prospective multicenter studies with large imaging registries are needed to facilitate this work.

In conclusion, most adnexal lesions in pregnancy are benign and can be managed conservatively, with a significant proportion resolving spontaneously. Where appropriate, conservative management should be offered, with reassurance that intervention is rarely required in the absence of symptoms or suspicious imaging features.

DEs continue to pose a diagnostic challenge owing to their morphological overlap with BOTs, however this review offers more objective descriptions of their morphology and a program of surveillance for DEs based on both the literature and our experience. While diagnostic models, such as the IOTA two‐step strategy, ADNEX and Simple Rules models, demonstrate high specificity and NPV, their clinical utility is limited in this population owing to frequent misclassification of decidualized lesions as malignant.

Until pregnancy‐specific models are validated, expert ultrasound assessment combined with longitudinal follow‐up remains the most reliable approach, with MRI as a second‐line strategy. In cases in which sonographic features raise concern for malignancy, timely referral to a tertiary gynecology–oncology center is warranted for diagnostic assessment and, if necessary, to initiate treatment during pregnancy in order to minimize treatment delay.

## Supporting information


**Appendix S1** Search strategy.

**Appendix S2** Excluded studies and reasons for exclusion.

**Table S1** Management outcomes, type of surgery performed and timing of surgery in patients with adnexal lesion in pregnancy.
**Table S2** Tumour subtypes observed in each study.
**Table S3** Illustrative table of adnexal lesions arising in pregnancy.
**Table S4** Imaging features of benign lesions.
**Table S5** Imaging features of decidualized endometriomas.
**Table S6** Imaging features of borderline and malignant ovarian tumors.
**Table S7** Diagnostic approach, descriptive outcomes and performance of models used.

**Figure S1** Bar chart showing histological subtypes of benign (a), borderline (b) and malignant (c) tumors.

**Figure S2** Bar chart showing aggregate risk‐of‐bias assessment scores.

## Data Availability

Research data are not shared.
