# Supplementary material for: Diagnostic imaging of adnexal lesions in pregnancy: systematic review
Source: Ultrasound Obstet Gynecol. 2026 Aug 10;68(4):480–91. doi: 10.1002/uog.70296 (PMC13641726; doi:10.1002/uog.70296)
Supplement: Supplementary file 1 [file UOG-68-480-s002.docx]

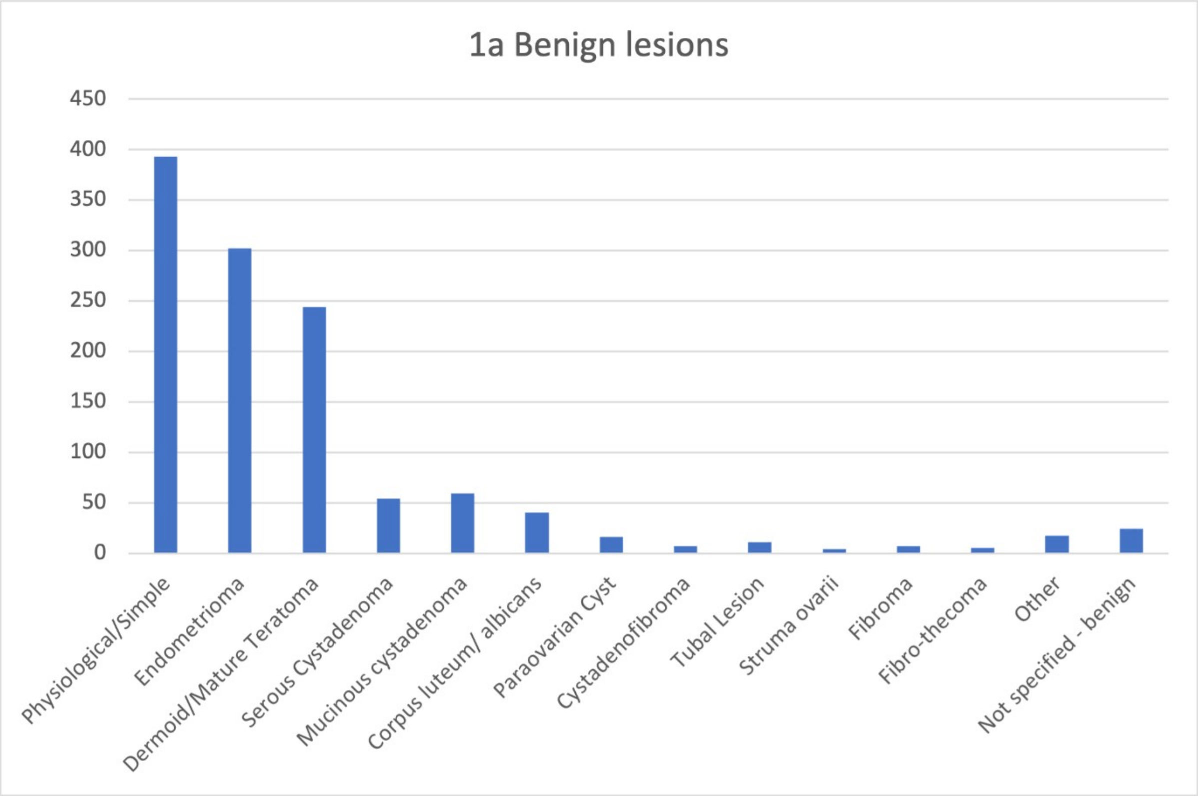
1a Benign lesions
450
400
350
300
250
200
150
100
50
0
Physiological/Simple
Endometrioma
Dermoid/Mature Teratoma
Serous Cystadenoma
Mucinous cystadenoma
Corpus luteum/ albicans
Paraovarian Cyst
Cystadenofibroma
Tubal Lesion
Struma ovarii
Fibroma
Fibro-thecoma
Other
Not specified - benign

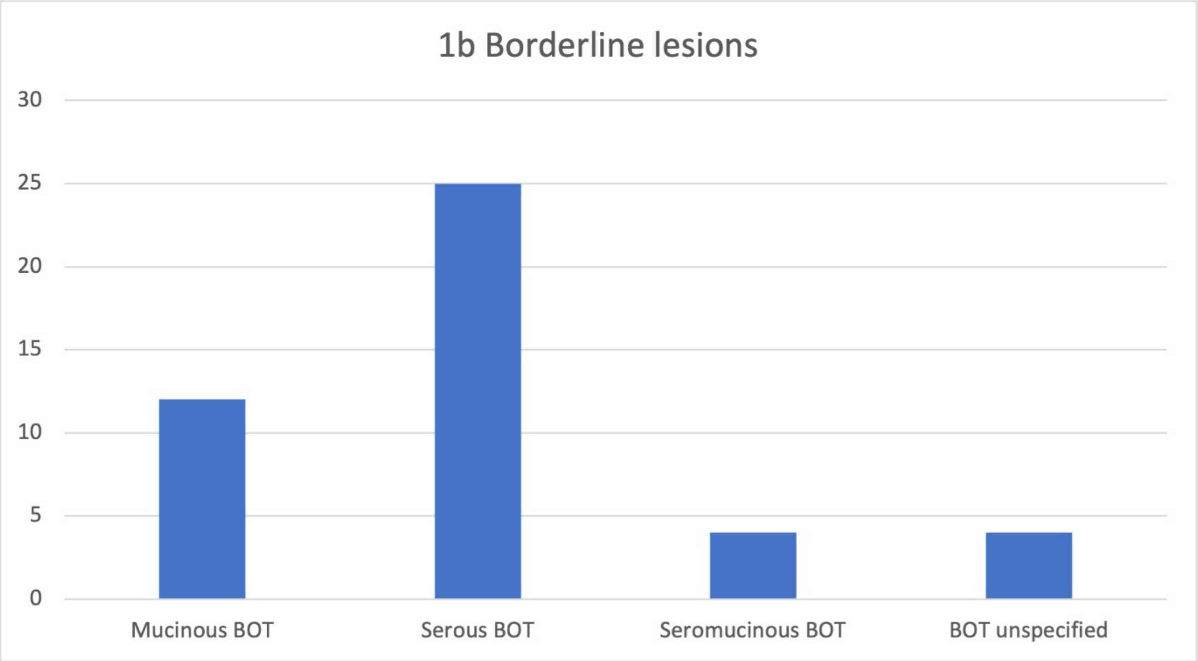
1b Borderline lesions
30
25
20
15
10
5
0
Mucinous BOT
Serous BOT
Seromucinous BOT
BOT unspecified

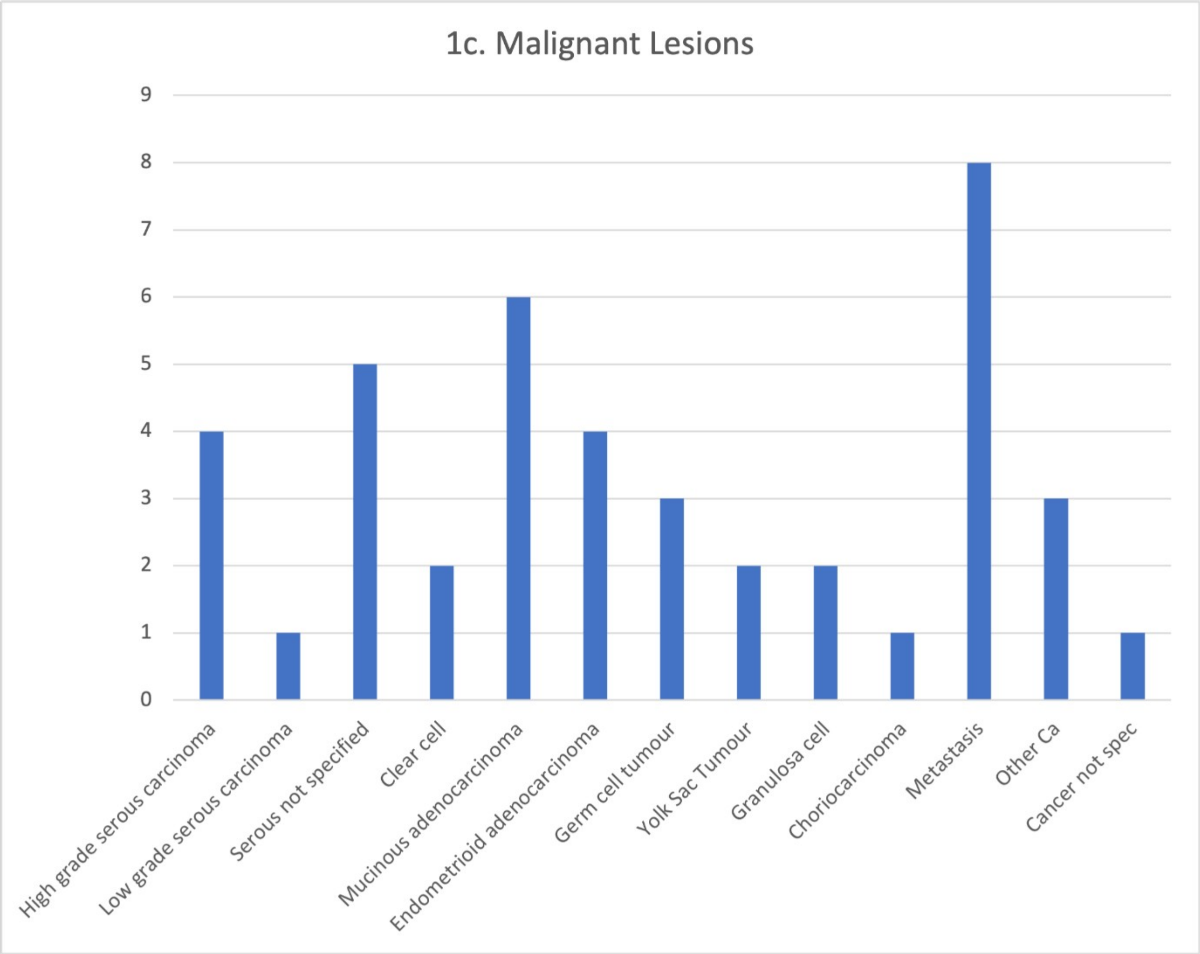

1c. Malignant Lesions
9
8
7
6
5
4
3
2
1
0
High grade serous carcinoma
Low grade serous carcinoma
Serous not specified
Clear cell
Mucinous adenocarcinoma
Endometrioid adenocarcinoma
Germ cell tumour
Yolk Sac Tumour
Granulosa cell
Choriocarcinoma
Metastasis
Other Ca
Cancer not spec
