# Supplementary material for: Diagnostic imaging of adnexal lesions in pregnancy: systematic review
Source: Ultrasound Obstet Gynecol. 2026 Aug 10;68(4):480–91. doi: 10.1002/uog.70296 (PMC13641726; doi:10.1002/uog.70296)
Supplement: Supplementary file 1 [file UOG-68-480-s001.docx]

**CURRENT EVIDENCE FOR PRE-OPERATIVE RISK ASSESSMENT OF OVARIAN MASSES IN PREGNANCY: SEARCH STRATEGY USING MESH TERMS**

**Pre-Operative Care and Risk Assessment:**

("Preoperative Care"[MeSH] OR "Perioperative Care"[MeSH]) AND "Risk Assessment"[MeSH])

**Ovarian Masses:**

("Ovarian Neoplasms"[MeSH] OR "Ovarian Cysts"[MeSH])

**Pregnancy:**

("Pregnancy"[MeSH] OR "Pregnancy Complications, Neoplastic"[MeSH] OR "Pregnancy Trimester, First"[MeSH] OR "Pregnancy Trimester, Second"[MeSH] OR "Pregnancy Trimester, Third"[MeSH])

**Imaging and Diagnostic Methods:**

("Ultrasonography, Prenatal"[MeSH] OR "Magnetic Resonance Imaging"[MeSH] OR "Tomography, X-Ray Computed"[MeSH] OR "Diagnostic Imaging"[MeSH])

**Other Relevant Terms:**

("Biomarkers, Tumor"[MeSH] OR "Diagnosis, Differential"[MeSH] OR "Gynecologic Surgical Procedures"[MeSH] OR "Laparoscopy"[MeSH] OR "Neoplasm Staging"[MeSH] OR "Prognosis"[MeSH] OR "Follow-Up Studies"[MeSH] OR "Incidental Findings"[MeSH] OR "Clinical Decision-Making"[MeSH] OR "Patient Safety"[MeSH])

**CURRENT EVIDENCE FOR PRE-OPERATIVE RISK ASSESSMENT OF OVARIAN MASSES IN PREGNANCY: SEARCH STRATEGY USING NON-MESH TERMS**

*Pre-operative care and risk assessment:*

"Preoperative Care"; "Perioperative Care"; "Risk Assessment"

*Ovarian Masses:*

“Ovarian Tumo?R”; “Tumo?R Of The Ovary”; “Ovarian Cancer”; “Cancer Of The Ovary”; “Ovarian Cyst”; “Cyst Of The Ovary”; “Ovarian Pathology”; “Pathology Of The Ovary”; “Ovarian Neoplasm”; “Neoplasm Of The Ovary”; “Ovarian Malignancy”; “Malignancy Of The Ovary”; “Ovarian Mass”; “Mass Of The Ovary”

“Adnexal Tumo?R”; “Tumo?R Of The Adnexa”; “Adnexal Cancer”; “Cancer Of The Adnexa”; “Adnexal Cyst”; “Cyst Of The Adnexa”; “Adnexal Pathology”; “Pathology Of The Adnexa”; “Adnexal Neoplasm”; “Neoplasm Of The Adnexa”; “Adnexal Malignancy”; “Malignancy Of The Adnexa”

“Borderline tumo?r”; ”Adenoma”; “Dermoid”; “Teratoma”; “Corpus luteum”; “Corpora”; “Endometrioma”; “Ovarian germ cell Tumo?r”; “Yolk sac Tumo?r”; “Dysgerminoma”; “Epithelial Cell Tumo?r”; “Sex Cord Stromal Tumo?r”; “Embryonal Cell Tumo?r”; “Mesenchymal Tumo?r”; “Cystadenocarcinoma”

*Pregnancy:*

“Pregnancy”; “Pregnant”; “Pregnancies”; “Trimester”; “Intra-partum”; “Antenatal”; “Gestation”; “Pre-partum”; “Gravid”; “Expectant”, “Gestational”; “Pregnancy Complications”

*Imaging and Diagnostic Methods:*

“Ultrasound”; “Ultrasonography”; “Transvaginal”; “Transabdominal”; “Computed Tomography”; “CT”; “Magnetic Resonance Imaging”; “MRI”; “MR”; “Biomarker”; “Biomarkers”; “Biochemical Marker”; “Biochemical Markers”; “Mathematical Models”; “Imaging”; “Imaging Model”; “Imaging Models”; “Clinical Model”; “Clinical Models”; “Diagnosis”; “Diagnostic Index”; “Diagnostic Indices”; “Diagnosis, Differential”
