# Supplementary material for: Diagnostic imaging of adnexal lesions in pregnancy: systematic review
Source: Ultrasound Obstet Gynecol. 2026 Aug 10;68(4):480–91. doi: 10.1002/uog.70296 (PMC13641726; doi:10.1002/uog.70296)
Supplement: Supplementary file 3 [file UOG-68-480-s004.docx]

**Supplementary Table 1:** Management outcomes, type of surgery performed and timing of surgery in patients with adnexal lesion in pregnancy

| **Author (Year)** | **Conservatively Managed** | | **Resolution Antenatally** | | **Surgical Management** | | **Surgery in Pregnancy** | | **Type of Surgery** |
| --- | --- | --- | --- | --- | --- | --- | --- | --- | --- |
|  | **n** | **%** | **n** | **%** | **n** | **%** | **n** | **%*** |  |
| Barcroft 2024^1^ | 212 | 72.9 | 64 | 22.0 | 56 | 19.2 | 4 | 7.1 | CS + cystectomy (n = 4)  Surgery <12 weeks postpartum (n = 11)  Surgery >12 weeks postpartum (n = 37) |
| Bean 2023^2^ | 34 | 100.0 | 10 | 29.4 | 0 | 0.0 | 0 | 0.0 | - |
| Bromley 1997^3^ | 24 | 18.3 | 13 | 9.9 | 107 | 81.7 | 27 | 25.2 | 24 laparotomies in 2nd trimester, 3 caesaran cystectomies |
| Czekierdowski 2021^4^ | 0 | 0.0 | 0 | 0.0 | 36 | 100.0 | 36 | 100.0 | CS + tumorectomy (n = 20)  Antenatal surgery (n = 15)  Tumour biopsy (n = 1) |
| Condous 2004^5^ | 159 | 95.8 | 119 | 71.7 | 7 | 4.2 | 7 | 100.0 | CS + cystectomy (n = 3)  Laparotomy + USO at 14 weeks (n = 2)  Cyst aspiration (n = 1)  SMM + laparoscopic cystectomy (n = 1) |
| Gaughran 2024^6^ | 260 | 92.2 | 177 | 62.8 | 14 | 5.0 | 8 | 57.1 | Antenatal surgery (n = 2)  CS + cystectomy (n = 6) |
| Groszmann 2014^7^ | 0 | 0.0 | 0 | 0.0 | 22 | 100.0 | 0 | 0.0 | Not clear if all surgeries performed antenatally/at caesarean |
| Lee 2021^8^ | 0 | 0.0 | 0 | 0.0 | 234 | 99.1 | 223 | 95.2 | Benign Lesions:  Antenatal surgery (n = 99)  Immediately after delivery (at CS) (n = 112)  After delivery (n = 10)  Malignant Lesions:  Antenatal surgery (n = 5)  Immediately after delivery (at CS) (n = 7)  After delivery (n = 1)  Mean gestation at surgery  13.9 +- 5 weeks (benign)  17.4 +- 8.2 weeks (malignant) |
| Mascilini 2014^9^ | 0 | 0.0 | 0 | 0.0 | 18 | 100.0 | 18 | 100.0 | CS + surgery (n = 5)  Antenatal surgery (n = 13) |
| Orlov 2025^10^ | 56 | 98.2 | 6 | 10.5 | 1 | 1.8 | 1 | 100.0 | Antenatal (n = 1) |
| Pateman 2014^11^ | 33 | 97.1 | 0 | 0.0 | 1 | 2.9 | 1 | 100.0 | CS + cystectomy (n = 1) |
| Rabiej-Wronska 2022^12^ | 67 | 50.0 | 0 |  | 61 | 45.5 | 50 | 82.0 | Antenatal surgery (n = 36)  CS + surgery (n = 14)  After delivery (n = 11) |
| Takeuchi 2016^13^ | 8 | 88.9 | 0 | 0.0 | 1 | 11.1 | 1 | 100.0 | Not stated |
| Testa 2021^14^ | 48 | 42.5 | 8 | 7.1 | 67 | 59.3 | 65 | 97.0 | CS + surgery (n = 18)  Antenatal surgery (n = 42)  Post-partum/TOP (n = 5)  Laparoscopy (n = 29), laparotomy (n = 45, inc. CS) |
| Zanetta 2003^15^ | 68 | 88.3 | 42 | 54.5 | 23 | 29.9 | 4 | 17.4 | Antenatal (n = 4) |

*n/N = number of those surgically managed during pregnancy as a proportion of all surgically managed lesions

**Lesions captured by paper by *Testa et al*^14^

Supplementary Table 2: Tumour subtypes observed in each study

| **Author Name** |  | **Total (n = 1506)** | **Barcroft 2024^1^** | | **Mascilini 2014^9^** | **Condous 2004^5^** | **Czekierdowski 2021^4^** | **Gaughran 2024^6^** | **Bean 2023^2^** | **Lee 2021^8^** | **Rabiej-Wronska 2022^12^** | **Pateman 2014^11^** | **Orlov 2025^10^** | **Zanetta 2003^15^** | **Bromley 1997^3^** | **Testa 2021^16^** |
| --- | --- | --- | --- | --- | --- | --- | --- | --- | --- | --- | --- | --- | --- | --- | --- | --- |
| **No. lesions (n)** |  |  | **291** | | **18** | **166** | **36** | **274** | **34** | **236** | **64** | **34** | **57** | **77** | **106** | **113** |
| Physiological/  ‘Simple’ | n | **393** |  | |  | 119 |  | 208 |  |  | 10 |  |  | 39 | 14 |  |
|  | *%* | ***26.1*** |  | |  | *71.7* |  | *75.9* |  |  | *15.6* |  |  | *68.4* | *18.2* |  |
| Endometrioma | n | **302** | 61 | | 18 | 7 | 11 | 19 | 34 | 32 | 7 | 34 | 57 |  | 16 | 6 |
|  | *%* | ***20.1*** | *21.0* | | *100.0* | *4.2* | *30.6* | *6.9* | *100.0* | *11.7* | *10.9* | *100.0* | *100.0* |  | *15.1* | *5.3* |
| Dermoid/  mature teratoma | n | **244** | 22 | |  | 16 | 11 | 25 |  | 88 | 19 |  |  | 11 | 40 | 12 |
|  | *%* | ***16.2*** | *7.6* | |  | *9.6* | *30.6* | *9.1* |  | *37.3* | *29.7* |  |  | *14.3* | *37.7* | *10.6* |
| Serous Cystadenoma | n | **54** | 12 | |  | 21 | 2 |  |  | 15 |  |  |  |  | 3 | 1 |
|  | *%* | ***3.6*** | *4.1* | |  | *12.7* | 5.6 |  |  | *6.4* |  |  |  |  | *2.8* | *0.9* |
| Mucinous cystadenoma | n | **59** |  | |  | 2 |  |  |  | 36 | 8 |  |  |  | 10 | 3 |
|  | *%* | ***3.9*** |  | |  | *1.2* |  | *0.0* |  | *15.3* | *12.5* |  |  |  | *9.4* | *2.7* |
| Corpus luteum/ albicans | n | **40** | 1 | |  |  |  |  |  | 39 |  |  |  |  |  |  |
|  | *%* | ***2.7*** | *0.3* | |  |  |  |  |  | *16.5* |  |  |  |  |  |  |
| Paraovarian Cyst | n | **16** |  | |  | 0 |  | 7 |  | 7 | 2 |  |  |  |  |  |
|  | *%* | ***1.1*** |  | |  | *0.0* |  | *2.6* |  | *3.0* | *3.1* |  |  |  |  |  |
| Cystadenofibroma | n | **7** |  | |  |  |  |  |  |  | 2 |  |  |  | 2 | 3 |
|  | *%* | ***0.5*** |  | |  |  |  |  |  |  | 0.03125 |  |  |  | 1.9 | 2.7 |
| Tubal Lesion | n | **11** | 2 | |  |  |  |  |  |  |  |  |  |  | 9 |  |
|  | *%* | ***0.7*** | 0.7 | |  |  |  |  |  |  |  |  |  |  | 8.5 |  |
| Struma ovarii | n | **4** | 2 | |  |  |  |  |  |  |  |  |  |  | 2 |  |
|  | *%* | ***0.3*** | *0.7* | |  |  |  |  |  |  |  |  |  |  | 1.9 |  |
| Fibroma | n | **7** | 1 | |  |  | 1 | 1 |  | 2 | 2 |  |  |  |  |  |
|  | *%* | ***0.5*** | *0.3* | |  | *0.0* | *2.8* | *0.4* |  | *0.8* | *3.1* |  |  |  |  |  |
| Fibro-thecoma | n | **5** |  | |  |  | 2 |  |  |  | 2 |  |  |  | 1 |  |
|  | *%* | ***0.3*** |  | |  |  | *5.6* |  |  |  | *3.1* |  |  |  | *0.9* |  |
| Other | n | **17** | 1 | |  |  |  | 3 |  | 4 |  |  |  |  | 8 | 1 |
|  | *%* | ***1.1*** | *0.3* | |  | *0.0* |  | *1.1* |  | *1.7* |  |  |  |  | *7.5* | *0.9* |
| Not specified - benign | n | **24** |  | |  |  |  |  |  |  |  |  |  | 24 |  |  |
|  | *%* | ***1.6*** |  | |  |  |  |  |  |  |  |  |  | *31.2* |  |  |
| Mucinous BOT | n | **12** | 4 | |  |  |  | 2 |  |  | 1 |  |  |  |  | 5 |
|  | *%* | ***0.8*** | *1.4* | |  |  |  | *0.7* |  |  | *1.6* |  |  |  |  | *4.4* |
| Serous BOT | n | **25** | 6 | |  |  | 2 |  |  |  | 3 |  |  |  |  | 14 |
|  | *%* | ***1.7*** | *2.1* | |  |  | *5.6* |  |  |  | *4.7* |  |  |  |  | *12.4* |
| Seromucinous BOT | n | **4** | 1 | |  |  |  |  |  |  | 2 |  |  |  |  | 1 |
|  | *%* | ***0.3*** | *0.3* | |  |  |  |  |  |  | *3.1* |  |  |  |  | *0.9* |
| BOT unspecified | n | **4** |  | |  | 1 |  |  |  |  |  |  |  | 3 |  |  |
|  | *%* | ***0.3*** |  | |  | *0.6* |  |  |  |  |  |  |  | *3.9* |  |  |
| High grade serous adenocarcinoma | **n** | **4** | |  |  |  |  |  |  |  |  |  |  |  |  | 4 |
|  | ***%*** | **0.3%** | |  |  |  |  |  |  |  |  |  |  |  |  | *3.5* |
| Low grade serous adenocarcinoma | **n** | **1** | |  |  |  |  |  |  |  |  |  |  |  |  | 1 |
|  | ***%*** | **0.07** | |  |  |  |  |  |  |  |  |  |  |  |  | *0.9* |
| Serous not specified | **n** | **5** | |  |  |  | 3 |  |  | 1 | 1 |  |  |  |  |  |
|  | ***%*** | **0.3** | |  |  |  | *8.3* |  |  | *0.4* | *1.6* |  |  |  |  |  |
| Clear cell | **n** | **2** | |  |  |  |  |  |  | 1 |  |  |  |  |  | 1 |
|  | ***%*** | **0.1** | |  |  |  |  |  |  | *0.4* |  |  |  |  |  | *0.9* |
| Mucinous adenocarcinoma | **n** | **6** | |  |  |  |  |  |  | 4 | 1 |  |  |  |  | 1 |
|  | ***%*** | **0.4** | |  |  |  |  |  |  | *1.7* | *1.6* |  |  |  |  | *0.9* |
| Endometrioid adenocarcinoma | **n** | **4** | |  |  |  | 1 |  |  |  |  |  |  |  |  | 3 |
|  | ***%*** | **0.3** | |  |  |  | *2.8* |  |  |  |  |  |  |  |  | *2.7* |
| Germ cell tumour | **n** | **3** | |  |  |  |  |  |  | 3 |  |  |  |  |  |  |
|  | ***%*** | **0.2** | |  |  |  |  |  |  | *1.3* |  |  |  |  |  |  |
| Yolk Sac Tumour | **n** | **2** | |  |  |  |  |  |  |  | 2 |  |  |  |  |  |
|  | ***%*** | **0.1** | |  |  |  |  |  |  |  | *3.1* |  |  |  |  |  |
| Granulosa cell | **n** | **2** | |  |  |  |  |  |  | 1 |  |  |  |  |  | 1 |
|  | ***%*** | **0.1** | |  |  |  |  |  |  | *0.4* |  |  |  |  |  | *0.9* |
| Choriocarcinoma | **n** | **1** | |  |  |  |  |  |  |  |  |  |  |  |  | 1 |
|  | ***%*** | **0.07** | |  |  |  |  |  |  |  |  |  |  |  |  | *0.9* |
| Metastasis | **n** | **8** | |  |  |  | 2 |  |  | 1 | 1 |  |  |  |  | 4 |
|  | ***%*** | **0.5** | |  |  |  | *5.6* |  |  | *0.4* | *1.6* |  |  |  |  | *3.5* |
| Other Ca | **n** | **3** | |  |  |  |  |  |  | 2 | 1 |  |  |  |  |  |
|  | ***%*** | **0.2** | |  |  |  |  |  |  | *0.8* | *1.6* |  |  |  |  |  |
| Cancer not spec | **n** | **1** | |  |  |  |  |  |  |  |  |  |  |  | 1 |  |
|  | ***%*** | **0.07** | |  |  |  |  |  |  |  |  |  |  |  | *0.8* |  |

**Supplementary table 3**: Illustrative table of ovarian lesions arising in pregnancy

| **Ovarian Lesion** | **Typical Imaging Features** | **Example Image** |
| --- | --- | --- |
| ***Common Benign Lesions*** | | |
| Serous cystadenoma | Unilocular, anechoic content, smooth internal cyst walls, colour score of 1 |   Benign cystadenoma identified at 13 weeks’ gestation, appearing as a unilocular lesion with anechoic content, smooth cyst walls and a colour score of 1. |
| Benign teratoma (dermoid cyst) | Unilocular, mixed echogenicity and acoustic shadows, colour score of 1 |   Mature teratoma seen at 30 weeks’ gestation, located in the right upper quadrant. It is unilocular with mixed echogenicity and acoustic shadows. |
| Mucinous cystadenoma | Unilocular, low-level content, smooth internal cyst walls, colour score of 1 |   Mucinous cystadenoma identified at 12 weeks’ gestation, appearing as a unilocular lesion with low level echogenicity. |
|  |  |  |
| *Cystadenofibroma* | Unilocular-solid or multi-locular solid, anechoic or low-level content, often avascular solid components, acoustic shadows |   Cystadenofibroma incidentally identified at 8 weeks’ gestation, likely serous, presenting as a multilocular lesion, with smooth internal cyst walls and anechoic content.    Lesion identified at 17 weeks’ gestation, histologically confirmed as a benign mucinous cystadenofibroma, presenting as a multilocular lesion, with smooth internal cyst walls and low level content. |
| ***Malignant and Borderline Lesions*** | | |
| *Serous Borderline Ovarian Tumour* | Unilocular-solid, anechoic content, irregular cyst walls with solid, irregular and vascular papillations |   Histologically confirmed serous borderline ovarian tumour, incidentally identified at 12 weeks’ gestation at TAUS, presenting as a unilocular-solid lesion, with >4 vascular and papillations with irregular margins.      Histologically confirmed serous borderline ovarian tumour incidentally identified at 6 weeks’ on TVUS, presenting as a unilocular-solid lesion with groundglass content, irregular cyst walls and >4 papillations with craggy appearances. |
| *Mucinous Borderline Ovarian Tumour* | Multilocular or multilocular-solid, low-level or groundglass content, ‘honeycomb’ appearance, may have irregular cyst walls with vascular papillations |   Histologically confirmed mucinous borderline ovarian tumour, incidentally identified at 6 weeks’, presenting as a multilocular-solid lesion with low-level and groundglass content, and vascular irregular solid papillations.    Histologically confirmed mucinous borderline ovarian tumour, incidentally identified at routine 20 week anomaly scan, presenting as a multilocular lesion with >10 cyst locules and a typical ‘honeycomb’ appearance, with low-level and anechoic content. |
| *Seromucinous Borderline Ovarian Tumour* | Unilocular-solid or multilocular-solid, anechoic or low-level content, irregular cyst walls with solid, irregular and vascular papillations |   Seromucinous borderline ovarian tumour identified at 6 weeks’ gestation, presenting as a multilocular-solid lesion with groundglass echogenicity and >4 vascular papillations. |
| *Serous carcinoma* | Vascular unilocular-solid, multilocular-solid or solid, with or without papillary projections. May be associated with ascites and omental disease in advanced or high grade disease. |     Histologically confirmed serous ovarian carcinoma (stage Ia) incidentally identified at the 11-14 week dating scan. Unilocular-solid with large, vascular and irregular papillary projections.  *Courtesy of Dr Natalia Rosello.*      A histologically confirm high grade serous ovarian cancer, presenting with a solid, vascular ovarian tumour at 10 weeks gestation.  *Courtesy of Prof. Frederic Amant and Dr Charlotte Lejeune.* |
| *Endometrioid Adenocarcinoma* | Unilocular-solid, multilocular-solid or solid, often with anechoic or low-level content, large vascular papillations or solid components |   Endometrioid adenocarcinoma (stage II) identified at routine 11-14 week scan. This presented as a large bilocular-solid lesion with large vascular, irregular papillations. |
| *Malignant Ovarian Germ Cell Tumour* | Often solid or multilocular-solid and highly vascular, often unilateral*  *dysgerminomas often bilateral and have ‘lobulated’ appearances |     Stage IIIc, Mixed malignant germ cell tumour (60% yolk sac tumour and 40% dysgerminoma) incidentally diagnosed in the third trimester of pregnancy. This presented as a large, vascular, solid tumour. |
| *Clear Cell Carcinoma* | Usually unilateral, unilocular-solid or multilocular-solid lesion, with groundglass content, vascular papillations. Often associated with underlying endometriosis. |     Stage IV histologically confirmed clear cell carcinoma incidentally found at 20 weeks’ gestation, multilocular-solid with >4 papillations, measuring maximally 120mm. Visible of TV and TA US. |
| ***Endometriosis in Pregnancy*** | | |
| *Typical Endometrioma* | Unilocular, groundglass or low level content, smooth internal cyst walls, colour score of 1 |   Typical appearing endometrioma seen at 17 weeks’ gestation, presenting as a unilocular cyst with groundglass content, smooth internal cyst walls and a colour score of 1. |
| *Decidualised Endometrioma* | Unilocular-solid or multilocular-solid, groundglass or low-level content, smooth and rounded vascular papillations |  (a)   (b)  Decidualised endometrioma seen at 23 weeks’ gestation (a), then again postpartum (b). Note the smooth and rounded papillations seen in pregnancy, with significant vascularity. |
|  |  |    a. 9 weeks gestation      b. 14 weeks gestation      c. 18 weeks gestation      d. 29 weeks gestation (TA image)      e. 6 weeks postpartum   Evolution of a decidualised endometrioma through pregnancy and postpartum, note a single smooth and rounded vascular papillation arising during pregnancy and regressing postpartum. |
| *Decidualised extra-ovarian endometriosis* | Deep endometriotic nodules often enlarge and decidualise, becoming hyperechoic with increased vascularity in early–mid pregnancy, but typically regress or resolve later in gestation or postpartum. |     Decidualised endometrioma seen at 10 weeks’ gestation with visible rectovaginal deep infiltarating endometriosis    Decidualisation seen of plaque of endometriosis at 16 weeks gestation    Ongoing vascular appearances at 22 weeks, with loss of hypoechoic region      Postpartum resolution of decidualisation with endometrioma. There is residual free fluid and a plaque of endometriosis seen at torus with resolution of vascular region |

| **Ovarian Lesion** | **Typical Imaging Features** | **Example Image** |
| --- | --- | --- |
| ***Common Benign Lesions*** | | |
| Serous cystadenoma | Unilocular, anechoic content, smooth internal cyst walls, colour score of 1 |   Benign cystadenoma identified at 13 weeks’ gestation, appearing as a unilocular lesion with anechoic content, smooth cyst walls and a colour score of 1. |
| Benign teratoma (dermoid cyst) | Unilocular, mixed echogenicity and acoustic shadows, colour score of 1 |   Mature teratoma seen at 30 weeks’ gestation, located in the right upper quadrant. It is unilocular with mixed echogenicity and acoustic shadows. |
| Mucinous cystadenoma | Unilocular, low-level content, smooth internal cyst walls, colour score of 1 |   Mucinous cystadenoma identified at 12 weeks’ gestation, appearing as a unilocular lesion with low level echogenicity. |
|  |  |  |
| *Cystadenofibroma* | Unilocular-solid or multi-locular solid, anechoic or low-level content, often avascular solid components, acoustic shadows |   Cystadenofibroma incidentally identified at 8 weeks’ gestation, likely serous, presenting as a multilocular lesion, with smooth internal cyst walls and anechoic content.    Lesion identified at 17 weeks’ gestation, histologically confirmed as a benign mucinous cystadenofibroma, presenting as a multilocular lesion, with smooth internal cyst walls and low level content. |
| ***Malignant and Borderline Lesions*** | | |
| *Serous Borderline Ovarian Tumour* | Unilocular-solid, anechoic content, irregular cyst walls with solid, irregular and vascular papillations |   Histologically confirmed serous borderline ovarian tumour, incidentally identified at 12 weeks’ gestation at TAUS, presenting as a unilocular-solid lesion, with >4 vascular and papillations with irregular margins.      Histologically confirmed serous borderline ovarian tumour incidentally identified at 6 weeks’ on TVUS, presenting as a unilocular-solid lesion with groundglass content, irregular cyst walls and >4 papillations with craggy appearances. |
| *Mucinous Borderline Ovarian Tumour* | Multilocular or multilocular-solid, low-level or groundglass content, ‘honeycomb’ appearance, may have irregular cyst walls with vascular papillations |   Histologically confirmed mucinous borderline ovarian tumour, incidentally identified at 6 weeks’, presenting as a multilocular-solid lesion with low-level and groundglass content, and vascular irregular solid papillations.    Histologically confirmed mucinous borderline ovarian tumour, incidentally identified at routine 20 week anomaly scan, presenting as a multilocular lesion with >10 cyst locules and a typical ‘honeycomb’ appearance, with low-level and anechoic content. |
| *Seromucinous Borderline Ovarian Tumour* | Unilocular-solid or multilocular-solid, anechoic or low-level content, irregular cyst walls with solid, irregular and vascular papillations |   Seromucinous borderline ovarian tumour identified at 6 weeks’ gestation, presenting as a multilocular-solid lesion with groundglass echogenicity and >4 vascular papillations. |
| *Serous carcinoma* | Vascular unilocular-solid, multilocular-solid or solid, with or without papillary projections. May be associated with ascites and omental disease in advanced or high grade disease. |     Histologically confirmed serous ovarian carcinoma (stage Ia) incidentally identified at the 11-14 week dating scan. Unilocular-solid with large, vascular and irregular papillary projections.  *Courtesy of Dr Natalia Rosello.*      A histologically confirm high grade serous ovarian cancer, presenting with a solid, vascular ovarian tumour at 10 weeks gestation.  *Courtesy of Prof. Frederic Amant and Dr Charlotte Lejeune.* |
| *Endometrioid Adenocarcinoma* | Unilocular-solid, multilocular-solid or solid, often with anechoic or low-level content, large vascular papillations or solid components |   Endometrioid adenocarcinoma (stage II) identified at routine 11-14 week scan. This presented as a large bilocular-solid lesion with large vascular, irregular papillations. |
| *Malignant Ovarian Germ Cell Tumour* | Often solid or multilocular-solid and highly vascular, often unilateral*  *dysgerminomas often bilateral and have ‘lobulated’ appearances |     Stage IIIc, Mixed malignant germ cell tumour (60% yolk sac tumour and 40% dysgerminoma) incidentally diagnosed in the third trimester of pregnancy. This presented as a large, vascular, solid tumour. |
| *Clear Cell Carcinoma* | Usually unilateral, unilocular-solid or multilocular-solid lesion, with groundglass content, vascular papillations. Often associated with underlying endometriosis. |     Stage IV histologically confirmed clear cell carcinoma incidentally found at 20 weeks’ gestation, multilocular-solid with >4 papillations, measuring maximally 120mm. Visible of TV and TA US. |
| ***Endometriosis in Pregnancy*** | | |
| *Typical Endometrioma* | Unilocular, groundglass or low level content, smooth internal cyst walls, colour score of 1 |   Typical appearing endometrioma seen at 17 weeks’ gestation, presenting as a unilocular cyst with groundglass content, smooth internal cyst walls and a colour score of 1. |
| *Decidualised Endometrioma* | Unilocular-solid or multilocular-solid, groundglass or low-level content, smooth and rounded vascular papillations |  (a)   (b)  Decidualised endometrioma seen at 23 weeks’ gestation (a), then again postpartum (b). Note the smooth and rounded papillations seen in pregnancy, with significant vascularity. |
|  |  |    f. 9 weeks gestation      g. 14 weeks gestation      h. 18 weeks gestation      i. 29 weeks gestation (TA image)      j. 6 weeks postpartum   Evolution of a decidualised endometrioma through pregnancy and postpartum, note a single smooth and rounded vascular papillation arising during pregnancy and regressing postpartum. |
| *Decidualised extra-ovarian endometriosis* | Deep endometriotic nodules often enlarge and decidualise, becoming hyperechoic with increased vascularity in early–mid pregnancy, but typically regress or resolve later in gestation or postpartum. |     Decidualised endometrioma seen at 10 weeks’ gestation with visible rectovaginal deep infiltarating endometriosis    Decidualisation seen of plaque of endometriosis at 16 weeks gestation    Ongoing vascular appearances at 22 weeks, with loss of hypoechoic region      Postpartum resolution of decidualisation with endometrioma. There is residual free fluid and a plaque of endometriosis seen at torus with resolution of vascular region |

Supplementary Table 4: Imaging Features of Benign Lesions

|  | | | | | **Barcroft 2024^1^** | **Condous 2004^5^** | **Czekierdowski 2021^4^** | **Lee 2021^8^** | **Orlov 2025^10^** | **Pateman 2014^11^** | **Rabiej-Wronska 2022^12^** | **Testa 2021^16^** | | **Pooled Data** |
| --- | --- | --- | --- | --- | --- | --- | --- | --- | --- | --- | --- | --- | --- | --- |
| Tumour Type(s) | | | | | **Pooled histology** | **Pooled histology** | **Benign** | **Benign** | **Endometriomas (20 week gestation)** | **Endometriomas** | **Benign** | **Benign (expectantly managed)** | **Benign (surgically managed)** | **Benign Only** |
| n | | | | | 291 | 166 | 27 | 223 | 38 | 34 | 52 | 48 | 29 | 451 |
| Lesion diameter | | | | |  | | | | | | | | | |
| Median (mm) | | | | | 48 | 48 | 71 | 76.3 | 41 | 14cc |  | 59 | 89 | 71 |
| Minimum (mm) | | | | | 34 | 12 | 39 |  | 28 | 3cc |  | 15 | 23 | 15 |
| Maximum (mm) | | | | | 65.5 | 115 | 206 |  | 56 | 30cc |  | 132 | 266 | 266 |
| Lesion Type | | | | |  |  | | | | | | | | Total (n/223) |
| Unilocular | | | n | | 218 |  | 8 |  | 14 | 29 | 21 | 27 | 10 | 109 |
|  |  |  | *%* | | *74.9* |  | *29.6* |  | *36.8* | *85.3* | *40.4* | *56.3* | *34.5* | *48.9* |
| Unilocular-solid | | | n | | 27 |  | 13 |  | 13 |  | 8 | 13 | 7 | 54 |
|  |  |  | *%* | | *9.3* |  | *48.2* |  | *34.2* |  | *15.4* | *27.1* | *24.1* | *24.2* |
| Multilocular | | | n | | 29 |  | 2 |  | 9 | 1 | 11 | 5 | 6 | 34 |
|  |  |  | *%* | | *10.0* |  | *7.4* |  | *23.7* | *2.9* | *21.2* | *10.4* | *20.7* | *15.2* |
| Multilocular-solid | | | n | | 9 |  | 2 |  | 1 |  | 6 | 1 | 5 | 15 |
|  |  |  | *%* | | *3.1* |  | *7.4* |  | *2.6* |  | *11.5* | *2.1* | *17.2* | *6.7* |
| Solid | | | n | | 8 |  | 2 | 2 | 1 |  | 3 | 2 | 1 | 11 |
|  |  |  | *%* | | *2.7* |  | *7.4* | *0.9%* | *2.6* |  | *5.8* | *4.2* | *3.4* | *4.9* |
| Number of Locules | | | | |  |  | | | | | | | | Total (n/229) |
| ≤10 | | | n | | 289 |  |  | 212 |  |  |  | 6 |  | 218 |
|  |  |  | *%* | | *99.3* |  |  | *95.1* |  |  |  | *12.5* |  | *95.1* |
| >10 | | | n | | 2 |  |  | 11 |  |  |  | 0 |  | 11 |
|  |  |  | *%* | | *0.7* |  |  | *4.9* |  |  |  | *0.0* |  | *4.8* |
| Content | | | | |  |  |  |  |  |  |  |  |  | *Total (n/299)* |
| Anechoic | | | n | | 126 | 117 | 7 | 96 | 2 |  | 12 | 126 | 117 | 117 |
|  |  |  | *%* | | *43.3* | *70.5* | *25.9* | *43.0* | *5.4* |  | *23.1* | *43.3* | *70.5* | *39.1* |
| Low level | | | n | | 22 | 2 | 1 | 62 | 2 |  | 5 | 22 | 2 | 70 |
|  |  |  | *%* | | *7.60* | *1.2* | *3.7* | *27.8* | *5.4* |  | *9.6* | *7.6* | *1.2* | *23.4* |
| Ground-glass | | | n | | 58 | 7 | *6* |  | 32 | 30 | 6 | 58 | 7 | 74 |
|  |  |  | *%* | | *19.9* | *4.2* | *22.2* |  | *86.5* | *88.2* | *11.5* | *19.90* | *4.2* | *24.7* |
| Haemorrhagic | | | n | | 9 | 21 |  |  | 1 |  | 4 | 9 | 21 | 5 |
|  |  |  | *%* | | *3.10* | *12.7* |  |  | *2.7* |  | *7.7* | *3.1* | *12.7* | *1.7* |
| Mixed | | | n | | 9 | 16 | *11* |  |  |  | 22 | 9 | 16 | 33 |
|  |  |  | *%* | | *3.1* | *9.6* | *40.8* |  |  |  | *42.3* | *3.1* | *9.6* | *11.0* |
| Colour score | | |  |  | | | | | | | | | | *Total (n/153)* |
| 1 | | | n | | 239 |  | *13* |  |  |  | 37 | 30 | 14 | 94 |
|  |  |  | *%* | | 82.1% |  | *48.2* |  |  |  | 71.2% | 62.5% | 48.3% | *61.4* |
| 2 | | | n | | 45 |  | *3* |  |  |  | 7 | 14 | 7 | 31 |
|  |  |  | *%* | | 15.5% |  | *11.1* |  |  |  | 13.5% | 29.2% | 24.1% | *20.3* |
| 3 | | | n | | 4 |  | *9* |  |  |  | 5 | 3 | 7 | 24 |
|  |  |  | *%* | | 1.4% |  | *33.3* |  |  |  | 9.6% | 6.3% | 24.1% | *15.7* |
| 4 | | | n | | 3 |  | *2* |  |  |  | 0 | 1 | 1 | 4 |
|  |  |  | *%* | | 1.0% |  | *7.4* |  |  |  | 0.0% | 2.1% | 3.4% | *2.6* |
| Max solid component diameter | | | | | 14.5 |  | 18 | 7.4 | 23 |  |  | 26 | 35 | 23 |
| Min | | | | | 11 |  | *2* |  | 8 |  |  | 7 | 2 | 2 |
| Max | | | | | 21.5 |  | *77* |  | 48 |  |  | 132 | 134 | 134 |
|  |  | | | | | | | | | | | | | *n/349* |
| Presence of solid papillations | | n | | | 31 | 3 | 11 | 12 | 15 |  |  | 13 | 9 | *60* |
|  |  | *%* | | | *10.7* | *1.8* | *40.7* | *5.4* | *39.5* |  |  | *27.1* | *31.0* | *17.2* |
|  |  | | | | | | | | | | | | | *n/33* |
| 1 | | n | | | 5 |  | *3* |  |  |  |  | 5 | 3 | 11 |
|  |  | *% (n/N)* | | | *16.1* |  | *27.3* |  |  |  |  | 38.5 | 33.3 | 33.3 |
| 2 | | n | | | 2 |  | *2* |  |  |  |  |  |  | 2 |
|  |  | *% (n/N)* | | | *6.5* |  | *18.2* |  |  |  |  |  |  | 6.1 |
| 3 | | n | | | 2 |  | *2* |  |  |  |  |  |  | 2 |
|  |  | *% (n/N)* | | | *6.5* |  | *18.2* |  |  |  |  |  |  | 6.1 |
| >3 | | n | | | 4 |  | *4* |  |  |  |  | 6 | 5 | 15 |
|  |  | *% (n/N)* | | | *12.9* |  | *36.4* |  |  |  |  | *46.2* | *55.6* | 45.5 |
| Missing | | n | | |  |  |  |  |  |  |  | 2 | 1 | 3 |
|  |  | *% (n/N)* | | |  |  |  |  |  |  |  | *15.4* | *11.1* | 9.1 |
| Average height of maximum solid papillation | | | | |  |  | *12* |  |  |  |  | 9.8 | 9 | *9.8* |
| Min | | | | |  |  | *3* |  |  |  |  | 5 | 4 | *3* |
| Max | | | | |  |  | *42* |  |  |  |  | 28 | 16 | *28* |
| Presence of colour flow in papillation | | n/N | | | 10 |  | *7* |  |  |  |  | 11 | 7 | 25 |
|  |  | *%* | | | *32.3* |  | *63.6* |  |  |  |  | *84.6* | *77.8* | 41.7 |
| Presence of ascites | | n | | |  |  | 0 | 6 |  |  |  |  |  | *-* |
|  |  | *%* | | |  |  | *0* | *2.7* |  |  |  |  |  | *-* |

**Supplementary Table S5:** Imaging Features of Decidualized Endometriomas

|  | | | **Barcroft 2024^1^** | **Bean 2023^2^** | **Groszmann 2014^7^** | **Mascillini 2018^9^** | **Orlov 2025 (20 week gestation)^10^** | **Pateman 2014^11^** | **Pooled Data** |
| --- | --- | --- | --- | --- | --- | --- | --- | --- | --- |
| Study inclusion | | | All endometrioma | All endometrioma | Only decidualised endometrioma | Only decidualised endometrioma | Endometrioma only, data presented for 20 week scan, cyst content data only available for 36 | All endometrioma |  |
| Total endometriomas (n) | | | 60 | 34 | 22 | 18 | 38 | 34 | 206 |
| Not Decidualised | n | | 42 | 0 | 0 | 0 | 23 | 30 | 95 |
|  | *%* | | 70.0 | 0.0 | 0.0 | 0.0 | 60.5 | 88.2 | 46.1 |
| Decidualised | n | | 18 | 34 | 22 | 18 | 15 | 4 | 111 |
|  | *%* | | 30.0 | 100.0 | 100.0 | 100.0 | 39.5 | 11.8 | 53.9 |
| Proven Cancer | n | | 1 |  |  |  |  |  |  |
|  | *%* | | 1.7 |  |  |  |  |  |  |
| **Characteristics of Decidualised Endometriomas** | | | | | | | | | |
| Average Max Diameter (mm) | | | 48.1 | 29.5 | 73 | 66 | 41 | Median vol: 14cc | 48.1 |
| Minimum (mm) | | | 17 | 11.3 | 30 | 41 | 28 | 3cc | 11.3 |
| Maximum (mm) | | | 102 | 82.3 | 120 | 121 | 56 | 30cc | 121 |
| Cyst Type | | |  | | | | | | n/58 |
| Unilocular | | n | 3 |  | 2 | 1 |  |  | 6 |
|  |  | *%* | 16.70% |  | 9.10% | 5.60% |  |  | 10.3% |
| Unilocular-solid | | n | 11 |  | 10 | 10 |  |  | 31 |
|  |  | *%* | 61.10% |  | 45.50% | 55.60% |  |  | 53.4% |
| Multilocular | | n | 0 |  | 5 |  |  |  | 5 |
|  |  | *%* | 0.00% |  | 22.70% | 0.00% |  |  | 8.6% |
| Multilocular-solid | | n | 3 |  | 3 | 7 |  |  | 13 |
|  |  | *%* | 16.70% |  | 13.60% | 38.90% |  |  | 22.4% |
| Solid | | n | 1 |  | 2 |  |  |  | 3 |
|  |  | *%* | 5.60% |  | 9.10% | 0.00% |  |  | 5.2% |
| Number of locules | | |  | | | | | | |
| ≤10 | | n |  |  |  | 18 |  |  |  |
|  |  | *%* |  |  |  | 100.00% |  |  |  |
| >10 | | n |  |  |  | 0 |  |  |  |
|  |  | *%* |  |  |  | 0.00% |  |  |  |
| Content | | |  | | | | | | *Total (n/38)* |
| Anechoic | | n |  |  | 5 | 2 |  |  | 7 |
|  |  | *%* |  |  | 22.70% | 11.10% |  |  | *18.4* |
| Low level | | n |  |  | 7 | 4 |  |  | 11 |
|  |  | *%* |  |  | 31.80% | 22.20% |  |  | *28.9* |
| Groundglass | | n |  |  | 8 | 12 |  |  | 20 |
|  |  | *%* |  |  | 36.40% | 66.70% |  |  | *52.6* |
| Haemorrhagic | | n |  |  | 0 | *0* |  |  | 0 |
|  |  | *%* |  |  | 0.00% | 0.00% |  |  | *0.0* |
| Mixed | | n |  |  | 0 | *0* |  |  | 0 |
|  |  | *%* |  |  | 0.00% | 0.00% |  |  | *0.0* |
| Colour score | |  | | | | | | | *n/58* |
| 1 | | n | 5 |  | 5 | 3 |  |  |  |
|  |  | *%* | 27.80% |  | 22.70% | 16.70% |  |  | 13 |
| 2 | | n | 8 |  | 3 | 2 |  |  | 22.4% |
|  |  | *%* | 44.40% |  | 13.60% | 11.10% |  |  | 13 |
| 3 | | n | 3 |  | 7 | 13 |  |  | 22.4% |
|  |  | *%* | 16.70% |  | 31.80% | 72.20% |  |  | 23 |
| 4 | | n | 2 |  | 6 |  |  |  | 39.7% |
|  |  | *%* | 11.10% |  | 27.30% | 0.00% |  |  | 8 |
| Not stated | | n |  |  | 1 |  |  |  | 13.8% |
|  | | *%* |  |  | 4.50% |  |  |  | 1 |
| Average Max solid component diameter | | | 17 |  |  | 28 |  |  | 22.5 |
| Min | | | 6 |  |  | 6 |  |  | 6 |
| Max | | | 52 |  |  | 79 |  |  | 65.5 |
| Presence of solid papillations | n | | 14 |  | 14 | 17 |  |  | *45* |
|  | *%* | | 77.8 |  | 63.60 | 94.40 |  |  | *93.75 (n/58)* |
| Number of papillations |  | |  |  |  |  |  |  | *n/31* |
| 1 | n | | 10 |  |  | 4 |  |  | 14 |
|  | *% (n/N)* | | 71.40% |  |  | 23.50% |  |  | 45.2% |
| 2 | n | | 2 |  |  | 3 |  |  | 5 |
|  | *% (n/N)* | | 14.30% |  |  | 17.60% |  |  | 16.1% |
| 3 | n | | 1 |  |  | 6 |  |  | 7 |
|  | *% (n/N)* | | 7.10% |  |  | 35.30% |  |  | 22.6% |
| >3 | n | | 1 |  |  | 4 |  |  | 5 |
|  | *% (n/N)* | | 7.10% |  |  | 23.50% |  |  | 16.1% |
| Average height of maximum solid papillation | | |  |  |  | 18 |  |  |  |
| Min | | |  |  |  | 3 |  |  |  |
| Max | | |  |  |  | 33 |  |  |  |
| Presence of colour flow in papillation | n/N | | 8 |  | 13 | 17 |  |  | *38* |
|  | *%* | | 57.10% |  | 92.90% | 94.40% |  |  | *84.4 (n/45)* |
| Presence of ascites | n | |  |  |  | 0 |  |  |  |
|  | *%* | |  |  |  | 0.00% |  |  |  |

**Supplementary table 6:** Imaging Features of Borderline and Malignant Ovarian Tumours

*** Lee et al & Testa et al only, mixed dataset in Rabiej-Wronska et al and Cziekerdowski et al**

|  | | | **Testa 2021^14^** | **Lee 2021^8^** | **Czekierdowski 2021^4^** | **Rabiej-Wronska 2022^12^** | **Testa 2021^16^** | **Pooled*** |
| --- | --- | --- | --- | --- | --- | --- | --- | --- |
| Tumour Type(s) | | | **Serous, mucinous and seromucinous** | **Malignant only** | **Pooled Malignant + BOT** | **Pooled Malignant + BOT** | **Malignant only** | **Malignant Only** |
| n | | | 20 | 13 | 9 | 12 | 16 | 29 |
| Average (mm) | | | 88 | 115.8 | 90 |  | 115 | 115.4 |
| Minimum (mm) | | | 37 |  | 45 |  | 43 | 43 |
| Maximum (mm) | | | 216 |  | 135 |  | 182 | 182 |
| Unilocular | n | | 0 |  | 0 | 2 | 0 | 0 |
|  | *%* | | 0.0% |  | 0.0% | 16.7% | 0.0% | 0.00% |
| Unilocular-solid | n | | 9 |  | 2 | 3 | 2 | 2 |
|  | *%* | | 45.0% |  | 22.2% | 25.0% | 12.5% | 7.69% |
| Multilocular | n | | 4 |  | 0 | 0 | 0 | 0 |
|  | *%* | | 20.0% |  | 0.0% | 0.0% | 0.0% | 0.00% |
| Multilocular-solid | n | | 6 |  | 4 | 3 | 8 | 8 |
|  | *%* | | 30.0% |  | 44.5% | 25.0% | 50.0% | 30.77% |
| Solid | n | | 1 | 10 | 3 | 4 | 6 | 16 |
|  | *%* | | 5.0% | 76.9% | 33.3% | 33.3% | 37.5% | 61.54% |
| ≤10 | n | | 6 | 11 |  |  | 5 | 16 |
|  | *%* | | 60.0% | 84.6% |  |  | 31.3% | 76.19% |
| >10 | n | | 4 | 2 |  |  | 3 | 5 |
|  | *%* | | 40.0% | 15.4% |  |  | 18.8% | 23.81% |
| Anechoic | n | |  | 2 | 5 | 1 |  | 2 |
|  | *%* | |  | 15.4% | 55.6% | 8.3% |  | 33.3% |
| Low level | n | |  | 4 | 0 | 1 |  | 4 |
|  | *%* | |  | 40.0% | 0.0% | 8.3% |  | 66.6% |
| Groundglass | n | |  |  | 0 | 1 |  | 0 |
|  | *%* | |  |  | 0.0% | 8.3% |  | 0.0 |
| Haemorrhagic | n | |  |  |  | 2 |  | 0 |
|  | *%* | |  |  |  | 16.7% |  | 0.00% |
| Mixed | n | |  |  | 1 | 7 |  | 0 |
|  | *%* | |  |  | 11.1% | 58.3% |  | 0 |
| 1 | n | | 4 |  | 0 | 1 | 0 | 0 |
|  | *%* | | 20.0% |  | 0.0% | 8.3% | 0.0% | 0.0% |
| 2 | n | | 7 |  | 2 | 4 | 3 | 3 |
|  | *%* | | 35.0% |  | 22.2% | 33.3% | 18.8% | 18.8% |
| 3 | n | | 9 |  | 5 | 6 | 8 | 8 |
|  | *%* | | 45.0% |  | 55.6% | 50.0% | 50.0% | 50.0% |
| 4 | n | | 0 |  | 2 | 1 | 5 | 5 |
|  | *%* | | 0.0% |  | 22.2% | 8.3% | 31.3% | 31.3% |
| Max solid component diameter | | | 26.6 | 23.4 | 39 |  | 68.2 | 45.8 |
| Min | | | 7 |  | 17 |  | 17 | 17 |
| Max | | | 160 |  | 89 |  | 165 | 165 |
| Presence of solid papillations | | n | 15 | 2 | 5 |  | 6 | 8 |
|  |  | *%* | 75.0% | 8.5% | 55.6% |  | 37.5% | 27.59% |
| 1 | | n | 2 |  | 1 |  | 1 | 1 |
|  |  | *% (n/N)* | 13.3% |  | 11.1% |  | 16.7% | 16.70% |
| 2 | | n |  |  | 0 |  | 0 | 0 |
|  |  | *% (n/N)* |  |  | 0.0% |  | 0.0% | 0.00% |
| 3 | | n |  |  | 1 |  | 0 | 0 |
|  |  | *% (n/N)* |  |  | 11.1% |  | 0.0% | 0.00% |
| >3 | | n | 10 |  | 3 |  | 5 | 5 |
|  |  | *% (n/N)* | 66.7% |  | 33.3% |  | 83.3% | 83.30% |
| Missing | | n | 3 |  | 0 |  |  |  |
|  |  | *% (n/N)* | 20.0% |  | 0.0% |  |  |  |
| Average height of maximum solid papillation | | | 13.2 |  | 15 |  | 16.8 | 18.4 |
| Min | | | 5 |  | 11 |  | 9 | 9.5 |
| Max | | | 31 |  | 17 |  | 22 | 22 |
| Presence of colour flow in papillation | | n/N | 15 |  | 4 |  | 6 | 6 |
|  |  | *%* | 75.0% |  | 80.0% |  | 100.0% | 100.00% |
| Presence of ascites | | n |  | 1 | 1 |  |  | 1 |
|  |  | *%* |  | 7.7% | 11.1% |  |  | 7.70% |
| Stage | |  |  |  |  |  |  |  |
| 1a | | n |  |  |  |  | 2 | 2 |
|  |  | *%* |  |  |  |  | 12.5% | 18.2% |
| 1b | | n |  |  |  |  |  |  |
|  |  | *%* |  |  |  |  |  |  |
| 1c | | n |  |  |  |  | 4 | 4 |
|  |  | *%* |  |  |  |  | 25.0% | 36.3% |
| IIB | | n |  |  |  |  | 1 | 1 |
|  | | *%* |  |  |  |  | 6.3% | 9.0% |
| IIIB | | n |  |  |  |  |  |  |
|  | | *%* |  |  |  |  |  |  |
| IIIA | | n |  |  |  |  |  |  |
|  | | *%* |  |  |  |  |  |  |
| IIIC | | n |  |  |  |  | 3 | 3 |
|  | | *%* |  |  |  |  | 18.8% | 27.30% |
| IV | | n |  |  |  |  | 1 | 1 |
|  | | *%* |  |  |  |  | 6.3% | 9.1% |

**Supplementary Table 7:** Diagnostic approach, descriptive outcomes and performance of models used

| **Study** | **Imaging modality** | **Reference standard** | **Approach** | **Model Used** | **n** | **Sens** | **Spec** | **PPV** | **NPV** | **AUC** | **Descriptive Statistics** |
| --- | --- | --- | --- | --- | --- | --- | --- | --- | --- | --- | --- |
| Barcroft (2024)^1^ | Ultrasound | Expert opinion or histology | Diagnostic model | IOTA 2 step strategy (BDs + ADNEX) | 83 | 63.6 | 90.2 | 21.9 | 98.3 | - |  |
| Bean (2023)^2^ | Ultrasound | Expert opinion or histology | Longitudinal assessment | - | - | - | - | - | - | - |  |
| Bromley (1997)^3^ | Ultrasound | Histology | Descriptive | - | - | - | - | - | - | - | Cysts described as septate, simple or based on 'pattern recognition'.  89.3% of lesions could be classified during pregnancy as benign. 10.7% had features suspicious of malignancy. |
| Condous (2004)^5^ | Ultrasound | Expert opinion or histology | Descriptive | IOTA terms and definitions | - | - | - | - | - | - |  |
| Czekierdowski (2021)^4^ | Ultrasound | Histology | Diagnostic model | ROMA | 24 | 25.0 | 100.0 | 100.0 | 73.0 | - | Using the Simple Rules Risk, no malignancies were misclassified as benign. Data not available for 12 patients to apply ROMA. |
|  |  |  |  | IOTA Simple Rules Risk | 36 | 100.0 | 37.0 | 35.0 | 100.0 | - |  |
|  |  |  |  | ADNEX cut off >20% | 36 | 78.0 | 70.0 | 47.0 | 90.0 | - |  |
| Gaughran (2024)^6^ | Ultrasound | Expert opinion or histology | Diagnostic model | IOTA Simple Rules | NC | NC | NC | NC | NC | NC | IOTA Simple Rules applicable in 272/274 (99.3%) lesions |
| Groszmann (2014)^7^ | Ultrasound | Histology | Descriptive | - | - | - | - | - | - | - |  |
| Lee (2021)^8^ | Ultrasound | Histology | Diagnostic Model | Sassone | 236 | 69.23 | 84.75 | 20.9 | 97.9 | 0.831 |  |
|  |  |  |  | Lener | 236 | 76.92 | 68.61 | 12.5 | 98.1 | 0.71 |  |
|  |  |  |  | IOTA ADNEX | 236 | 61.54 | 84.75 | 19.0 | 97.4 | 0.709 |  |
|  |  |  |  | Combined model | 236 | 69.23 | 94.62 | 42.9 | 98.1 | 0.883 |  |
| Mascilini (2014)^9^ | Ultrasound | Histology | Descriptive | IOTA terms and definitions and expert opinion | 18 | - | 50 | - | 100 | - |  |
| Orlov (2025)^10^ | Ultrasound | Expert opinion | Longitudinal assessment | IOTA terms and definitions | - | - | - | - | - | - |  |
| Panico (2023) | MRI | Histology or Expert opinion | Diagnostic model | Non-contrast MRI score | 20 | 85.7 | 76.9 | 66.7 | 90.9 | - | Performance of NCMS score only available of non-expert radiologist |
| Pateman (2014)^11^ | Ultrasound | Expert opinion | Longitudinal assessment | - | - | - | - | - | - | - |  |
| Rabiej-Wronska (2022)^12^ | Ultrasound | Histology | Diagnostic model | IOTA Simple Rules - cut off 1 | 61 | 100 | 51.02 | 33.33 | 100 | - |  |
|  |  |  |  | IOTA Simple Rules - cut off 3 | 61 | 91.67 | 53.06 | 32.35 | 96.3 | - |  |
|  |  |  |  | IOTA Simple Rules - cut off 30 | 61 | 91.67 | 73.47 | 45.83 | 97.3 | - |  |
|  |  |  |  | IOTA Simple Rules - excluding inconclusive | 61 | 87.5 | 94.44 | 77.78 | 97.14 | - |  |
|  |  |  |  | IOTA Simple Rules - classify inconclusive as probably malignant | 61 | 91.67 | 69.39 | 42.31 | 97.14 | - |  |
| Takeuchi (2016)^13^ | MRI | Histology or resolution | Descriptive | - | - | - | - | - | - | - | T2 weighted images. Mural nodules exhibit high signal intensity on DWI 800 only, whereas cancer contained high intensity on both DWI1500 and 800. High signal intensity of decidualised endometriomas was caused by vacularised, oedematous decidualised tissue with a high AFC value whereas cancer had a high signal intensity due to restricted water diffusion, reflecting hypercellularity, with a relatively low ADC value. |
| Testa (2021)^14^ | Ultrasound | Histology | Descriptive | IOTA terms and definitions and expert opinion | - | - | - | - | - | - |  |
| Zanetta (2003)^15^ | Ultrasound | Subjective assessment or histology | Longitudinal assessment | - | - | - | - | - | - | - |  |

Only first author is given for each study. NC, not calculated. ROMA: risk of ovarian malignancy algorithm.

1.	Barcroft J, Pandrich M, Del Forno S, et al. Evaluating use of IOTA two‐step strategy to classify adnexal masses identified in pregnancy: pilot study. *Ultrasound in Obstetrics & Gynecology*. 2024;

2.	Bean E, Knez J, Setty T, et al. Natural history of endometriosis in pregnancy: ultrasound study of morphology of deep endometriosis and ovarian endometrioma. *Ultrasound in Obstetrics & Gynecology*. 2023;62(4):585–593.

3.	Bromley B, Benacerraf B. Adnexal masses during pregnancy: accuracy of sonographic diagnosis and outcome. *Journal of ultrasound in medicine*. 1997;16(7):447–452.

4.	Czekierdowski A, Stachowicz N, Smoleń A, et al. Sonographic assessment of complex ultrasound morphology adnexal tumors in pregnant women with the use of IOTA simple rules risk and ADNEX scoring systems. *Diagnostics*. 2021;11(3):414.

5.	Condous G, Khalid A, Okaro E, Bourne T. Should we be examining the ovaries in pregnancy? Prevalence and natural history of adnexal pathology detected at first‐trimester sonography. *Ultrasound in Obstetrics and Gynecology: The Official Journal of the International Society of Ultrasound in Obstetrics and Gynecology*. 2004;24(1):62–66.

6.	Gaughran J, Magee C, Mitchell S, Knight CL, Sayasneh A. Adnexal Masses in Pregnancy: A Single-Centre Prospective Observational Cohort Study. *Diagnostics*. 2024;14(19):2182.

7.	Groszmann Y, Howitt BE, Bromley B, Feltmate CM, Benacerraf BR. Decidualized endometrioma masquerading as ovarian cancer in pregnancy. *Journal of Ultrasound in Medicine*. 2014;33(11):1909–1915.

8.	Lee SJ, Kim Y-H, Lee M-Y, et al. Ultrasonographic evaluation of ovarian mass for predicting malignancy in pregnant women. *Gynecologic Oncology*. 2021;163(2):385–391.

9.	Mascilini F, Moruzzi C, Giansiracusa C, et al. Imaging in gynecological disease (10): clinical and ultrasound characteristics of decidualized endometriomas surgically removed during pregnancy. *Ultrasound in Obstetrics & Gynecology*. 2014;44(3):354–360.

10.	Orlov S, Sladkevicius P, Eckerdal IR, Jokubkiene L. Morphological changes of endometriomas during pregnancy and after delivery detected using ultrasound. *Fertility and Sterility*. 2024;

11.	Pateman K, Moro F, Mavrelos D, Foo X, Hoo W-L, Jurkovic D. Natural history of ovarian endometrioma in pregnancy. *BMC Women's Health*. 2014;14:1–6.

12.	Rabiej-Wronska E, Wiechec M, Pitynski K, Wiercinska E, Kotlarz A. Ultrasound differentiation between benign versus malignant adnexal masses in pregnant patients. *Ginekologia Polska*. 2022;93(8):643–649.

13.	Takeuchi M, Matsuzaki K, Harada M. Computed diffusion-weighted imaging for differentiating decidualized endometrioma from ovarian cancer. *European Journal of Radiology*. 2016;85(5):1016–1019.

14.	Testa AC, Mascilini F, Quagliozzi L, et al. Management of ovarian masses in pregnancy: patient selection for interventional treatment. *International Journal of Gynecologic Cancer*. 2021;31(6)

15.	Zanetta G, Mariani E, Lissoni A, et al. A prospective study of the role of ultrasound in the management of adnexal masses in pregnancy. *BJOG: An International Journal of Obstetrics & Gynaecology*. 2003;110(6):578–583.

16.	Testa AC, Mascilini F, Quagliozzi L, et al. Management of ovarian masses in pregnancy: patient selection for interventional treatment. *International Journal of Gynecological Cancer*. 2021;31(6):899–906.
